# Coaxial Layered Fiber Spinning for Wind Turbine Blade Recycling

**DOI:** 10.1021/acssuschemeng.3c07484

**Published:** 2024-02-13

**Authors:** Varunkumar Thippanna, Arunachalam Ramanathan, Dharneedar Ravichandran, Abhinav Chavali, Barath Sundaravadivelan, Abhishek Saji Kumar, Dhanush Patil, Yuxiang Zhu, Rajesh Buch, Maryam Al-Ejji, Mohammad K. Hassan, Lindsay R. Bick, Martin Taylor Sobczak, Kenan Song

**Affiliations:** † Manufacturing Engineering, School of Manufacturing Systems and Networks (MSN), Ira A. Fulton Schools of Engineering, Arizona State University (ASU), Mesa, Arizona 85212, United States; ‡ Materials Science and Engineering, School for Engineering of Matter, Transport and Energy (SEMTE), Ira A. Fulton Schools of Engineering, Arizona State University (ASU), Tempe, Arizona 85287, United States; § Mechanical Engineering, The School of Engineering of Matter, Transport and Energy (SEMTE), Ira A. Fulton Schools of Engineering, Arizona State University (ASU), Tempe, Arizona 85287, United States; ∥ Rob and Melani Walton Sustainability Solutions Service, Arizona State University (ASU), Tempe, Arizona 85287, United States; ⊥ Center for Advanced Materials, Qatar University, P.O. BOX 2713,Doha 2713,Qatar; # Mechanical Engineering, College of Engineering, University of Georgia (UGA), 302 E. Campus Rd., Athens ,Georgia30602,United States; ∇ School of Manufacturing Systems and Networks (MSN), Ira A. Fulton Schools of Engineering, Arizona State University (ASU), Mesa, Arizona 85212, United States

**Keywords:** mechanical recycling, wind turbine blades, polymeric fibers, nanocomposites, sustainability

## Abstract

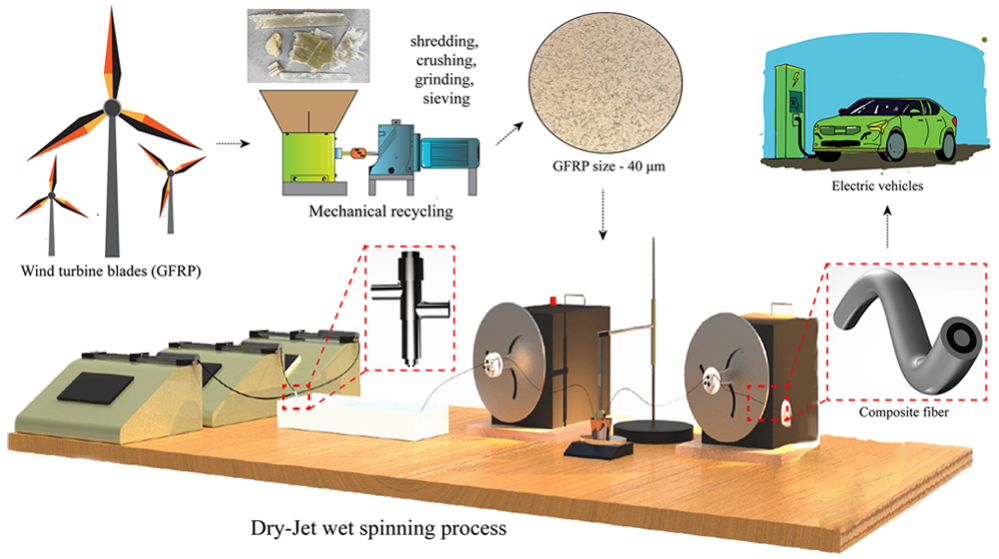

Plastics’ long degradation time and their role in adding millions of metric tons of plastic waste to our oceans annually present an acute environmental challenge. Handling end-of-life waste from wind turbine blades (WTBs) is equally pressing. Currently, WTB waste often finds its way into landfills, emphasizing the need for recycling and sustainable solutions. Mechanical recycling of composite WTB presents an avenue for the recovery of glass fibers (GF) for repurposing as fillers or reinforcements. The resulting composite materials exhibit improved properties compared to the pure PAN polymer. Through the employment of the dry-jet wet spinning technique, we have successfully manufactured PAN/GF coaxial-layered fibers with a 0.1 wt % GF content in the middle layer. These fibers demonstrate enhanced mechanical properties and a lightweight nature. Most notably, the composite fiber demonstrates a significant 24.4% increase in strength and a 17.7% increase in modulus. These fibers hold vast potential for various industrial applications, particularly in the production of structural components (e.g., electric vehicles), contributing to enhanced performance and energy efficiency.

## Abstract 2

Mechanical recycling of wind turbine blades yields high-strength PAN/GF coaxial-layered fibers through dry-jet wet spinning, promising enhanced performance in electric vehicle components.

## 1 Introduction

Plastic waste poses a global threat to nearly all freshwater and marine ecosystems. In 2016, it was estimated that aquatic environments received a staggering 19 to 23 million metric tons, constituting 11% of the world’s plastic waste. These figures are predicted to rise significantly, potentially reaching a staggering 53 million tons annually by 2030.^1^ Traditional methods of disposal, such as landfilling and incineration, exacerbate secondary contamination issues.^2,3^ To combat this crisis, we must prioritize recycling and more efficient waste management. By enhancing recycling technologies and infrastructure, we can significantly reduce the amount of plastic sent to landfills. Encouraging the use of biodegradable alternatives and responsible plastic consumption is equally vital. Raising awareness, implementing effective waste management practices, and driving policy initiatives are all essential components of the battle against plastic waste. A diverse array of recycling techniques have been explored to meet the demands of sustainable development and environmentally friendly practices, resulting in the creation of affordable, eco-friendly value-added products. Collaboration and concerted efforts are our path to a more sustainable future, one where we minimize the environmental impact of plastic waste.^4−6^ Policymakers are encouraging recycling and reuse to decrease resource consumption, waste generation, and the burden on landfills by reducing the need for raw materials and promoting sustainable practices.^7^ The lack of economic incentives for mechanical recycling, coupled with the complexities of recycling various plastics, has spurred the research community to develop technologies for closed-loop or open-loop recycling and upcycling, addressing the urgent issue of plastic pollution.^8,9^

The global wind energy market currently boasts a staggering 540 GW of installed wind turbine blade (WTB) capacity worldwide. As energy needs surge, the WTB size, weight, and strength rise accordingly. The expanding wind power sector fuels the continuous and rapid growth in the demand for WTBs.^10^ The development of effective processes for handling WTBs after their 20–25-year service life is of utmost importance in managing the climate crisis and mitigating the environmental consequences of the renewable energy source’s growth.^11^ Properly recycling of WTBs poses a significant challenge to environmental protection. Currently, the majority of WTB waste is disposed of in landfills. However, this is far from an environmentally sustainable solution, and many countries have prohibited the landfilling of composite waste. Recycling materials such as glass fiber-reinforced plastics (GFRP) used in WTBs is technically complex, making it challenging to convert them into valuable new materials. Finding effective recycling methods for WTBs is essential to address this environmental issue.^12−15^ Transforming waste from fiber-reinforced polymer composites into a valuable resource through a cradle-to-cradle approach is crucial for achieving endless material use and promoting a circular economy.^16^ The use of waste materials not only saves costs but also reduces the need for landfilling and incineration.^17^ To address plastic waste management issues in WTB disposal, it is vital to utilize existing recycling techniques, adopt a circular economy model, and explore sustainable and greener approaches for managing plastic waste from WTBs.^18^ Since the early 1990s, mechanical recycling has been extensively studied and proven to be an effective means of recycling GFRP. GFRP recycling techniques encompass three processes: mechanical-, thermal-, and chemical-based. Mechanical recycling, which is the most widely used and efficient method for reinforced composites, involves breaking down the composite material and reducing particle size through processes such as shredding, crushing, milling, and sieving.^19,20^ Mechanical recycling is efficient due to its low energy consumption and enables the recovery of GF, which consists of a polymer matrix and embedded glass fibers, without the use of hazardous solvents.^21^ The recycled GFRP waste powder and fibers, with distinct size gradings, can be incorporated into polymer applications as filler replacements or for the partial reinforcement of composite polymers. This approach offers a sustainable solution for their reuse.^22−24^

Particle-filled polymer composites are the subject of extensive research due to their desirable properties, which include being lightweight, cost-effective, durable, and tunable. Homogeneous mixing is commonly employed to achieve the isotropic properties. However, aligning particles within 1D polymer composite fibers has traditionally been limited to methods such as drawing or stretching. To obtain synergistic and hybrid properties, a combination of particle fillers and polymer matrices is used. This strategy is particularly valuable for creating composites with isotropic properties.^25,26^ Such composite fibers, incorporating particles, offer improved characteristics largely due to their high surface-to-volume ratio and compatibility with the matrix. One of the major challenges in preparing excellent composite fibers is achieving uniform dispersion of particles within the polymer matrix. This uniformity is essential for enhancing properties, such as flame retardancy, mechanical strength, barrier properties, and thermal conductivity. The ability to control these properties, especially their response to changes in temperature, is a significant advantage, unlocking the full potential of the material. Polymer molecules can interact with fillers, leading to the development of unique properties in composite systems. Ensuring the proper spatial orientation of filler particles is crucial for diverse applications of polymer–particle composites.^27−30^ For the production of commercial carbon fibers (CF), PAN-based fibers are favored. This preference is due to their high carbon yield, exceptional tensile strength, and rapid processing capabilities.^31^ To enhance the performance of polymers, they can be combined with various fillers, including glass fibers, metals, and particles, to create composites that allow control over mechanical properties, such as stiffness, strength, and toughness.^32,33^ Also, the coaxial-layered structure has found widespread use as a fiber geometry in various applications, including biomedical materials,^34^ drug delivery systems,^35^ strain sensors,^36^ and energy devices.^37^

In this investigation, we detail the production of PAN/PAN-GF/PAN coaxial-layered fibers utilizing the dry-jet wet-spinning technique. The process involves the passage of the polymer solution through a specially designed three-phase spinneret, followed by immersion in a methanol bath to ensure controlled solvent exchange. The obtained fibers are subsequently subjected to a drawing process in water and silicone oil at an elevated temperature to align the polymer and induce complete crystallization. The resulting fiber, which incorporates 0.1 wt % GF within the middle layer, demonstrates notable enhancements in mechanical properties. When compared to pure PAN fibers, this composite fiber exhibits a substantial 24.4% increase in strength and a noteworthy 17.7% increase in modulus.

## 2 Experimental Section

### 2.1 Materials

PAN copolymer (i.e., 99.5% acrylonitrile/0.5% methacrylate) with a molecular weight of 230,000 g/mol and a mean particle size of 50 μm was obtained from Goodfellow Cambridge Limited, Huntingdon England. Wind turbine blades containing GFRP were obtained from TPI Composites, Inc. (Iowa, US). Solvents, such as N,N-dimethylformamide (DMF) (ACS reagent, ≥99.8%) as a solvent to dissolve PAN and the media to disperse GF powder and methanol (ACS reagent, ≥99.8%) as the coagulant, were obtained from Sigma-Aldrich, US. All materials were purchased and used as received without further modifications.

### 2.2 Fiber Spinning and Post-Treatment

The following section describes the spinning of both one-phase and three-phase fibers.

#### 2.2.1 Preparation of One-Phase Feedstock

A PAN solution was made by dissolving 12 g of PAN in 100 mL of the DMF solvent, and the mixture was mechanically stirred at a temperature of 130 °C for 1 h until a transparent solution was obtained. To remove any trapped air, the solution was deaerated in a vacuum oven (Lindberg Blue M lab oven, Thermo Scientific US) for 30 min, ensuring a bubble-free solution. Subsequently, the solution was transferred into a metal syringe that was connected to a pump for the fiber-spinning process. The solution was injected into the spinneret at a controlled rate of 2 mL/min, facilitating the extrusion and formation of fibers.

#### 2.2.2 Preparation of Three-Layered PAN/PAN-GF/PAN Feedstock

**Figure 1:**
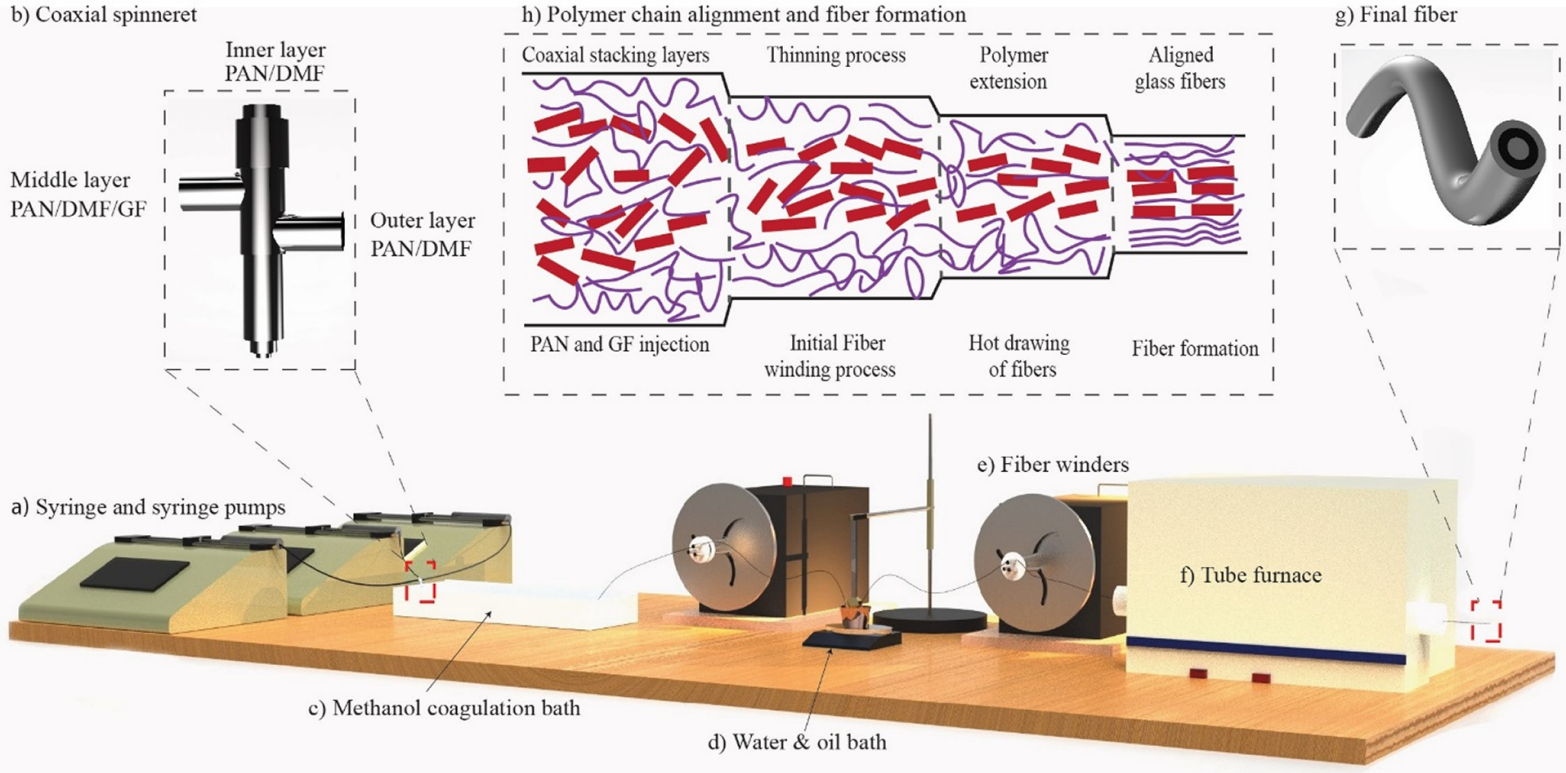
Manufacturing processes of fabricating three-layered composite fibers, namely, spinning, collection, coagulation, drawing, and post-treatment drying, with the unique setup of (a) material delivery via syringes and syringe pumps, (b) in-house designed spinneret, (c) methanol coagulation bath, (d) water and oil bath for temperature and antioxidation control via (e) fiber winders (effects on mechanical properties, Figures S2 and S3), (f) tube furnace for postdrawing treatment of (g) final fiber structure with layers, and (h) theoretical evolution of polymer chains during fiber spinning and drawing procedures (simulation effects on mechanical properties, Figure S4, with influences on thermal properties, in Figure S5; the layered structures can be seen in Figures S6–S8).

The three-layered fibers consist of a coaxial layer. The in-house-developed spinning setup (Figure 1a) includes the unique coaxial spinneret responsible for producing interior, middle, and exterior layers (Figure 1b). This unique coaxial spinneret was manufactured via the metal 3D printer, Concept Laser 2 using Inconel. Both interior and exterior layers were filled with 12 wt % PAN solutions (Table 1). In the middle layer, different GF filler concentrations (i.e., 0.1–200 wt % of GF with respect to PAN, as shown in Table 1) dispersed in 12 wt % PAN in the form of suspensions, which were obtained through tip sonication for 30 min at an amplitude of 60% (Q500, Fisher Scientific, US), with the same PAN/DMF solution preparation procedure as mentioned in the one-phase PAN fibers (Table 1). These GF fibers were mechanically recycled before the addition into the PAN/DMF solution through shredding, crushing, milling, and sieving (via the mesh 40). ImageJ was used to predict the average particle size of the process GFRP and was found to be 38 μm (Figure S1). All the solutions were transferred into the metal syringe(s) attached to the pumps (Figure 1a) and injected at rates of 2 mL/min for the interior, middle, and exterior layers to form the three-phase composite fibers.

**Table 1:** Summary of Fiber Type, Compositions, Processability and Testability, and Drawing Parameters

fiber type	terminology	compositions (wt %)			processability and testability			drawing results	
		interior and exterior layer PAN composition in DMF (wt %)	middle layer PAN wt % in DMF	middle layer (GF wt % w.r.t PAN)	spinnability	heat treatment	mechanical testability	total draw ratio	thickness (μm)
one-phase	PAN	12	12	0	Yes	fiber drawing at 85 °C in water and at 125, 135, and 145 °C in silicone oil before stabilization in tube furnace	yes	**32.50**	**83**
three-phase	PAN-0.1 wt % GF			0.1	Yes		yes	**63.00**	**54**
	PAN-1.0 wt % GF			1.0	Yes		yes	**41.00**	**65**
	PAN-2.5 wt % GF			2.5	Yes		yes	**43.50**	**60**
	PAN-10 wt % GF			10	Yes		yes	**31.50**	**78**
	PAN-50 wt % GF			50	Yes		yes	**17.00**	**83**
	PAN-100 wt % GF			100	Yes		yes	**28.00**	**144**
	PAN-200 wt % GF			200	Yes		yes	**28.00**	**224**
	10 wt % PAN/10 wt % PAN-200 wt % GF/10 wt % PAN	10	10	200	Yes		yes	**20.00**	**156**

#### 2.2.3 Fiber Spinning

During the fiber spinning process, the solution was injected in an air gap of 1.5–2.0 cm before entering the coagulation bath. The usage of an air gap in dry-jet wet spinning allowed fibers to undergo extension, reducing defect density and enabling molecular alignment. After immersing in the coagulation bath (Figure 1c), two diffusion processes occurred simultaneously, with (i) a polymer-rich phase condensed into the fiber and (ii) a solvent-rich phase (i.e., DMF) exchanging with the nonsolvent (i.e., methanol) to form the more solid-like gel fiber. As-spun fibers were soaked in methanol for 30 min. In the coagulation process, it is important for the coagulation rate to be sufficiently high to minimize the gradient between coagulated layers from the surface to the fiber core. Consistent coagulated structures prevented deformation toward the core, resulting in a circular shape.^38^ However, if the rates differed between layers, an irregular cross-sectional shape was likely to be formed due to diffusion mismatching and a solid polymer gradient. The chain alignment and dimensions of the as-spun fibers were also significantly influenced by the injection/flow rates. To reduce the fiber diameter and thus the defect density, higher flow rates through the coagulation bath with lower injection rates for the spinning dope were effective. However, a larger draw ratio (DR) (i.e., fiber drawing during coagulation) in the coagulant flow may not guarantee more polymer chain alignment due to stretching and recoiling processes. High flow rates can lead to drastic stretching and severe molecular recoil, hindering alignment; thus, our injection rates were optimized to be 2 mL/min and the winder collection rates were 33 m/min.

The following section describes the post-treatment of both one-phase and three-phase fibers. All as-spun fibers underwent a postprocessing procedure, which included a hot drawing and annealing process.

#### 2.2.4 Fiber Drawing

During the hot drawing stages, the fibers went through baths containing water and silicone oil to their maximum draw ratios before breakage (Figure 1d). During this process, the high shear force caused these macromolecules to align parallel to the fiber axis. Initially, fibers were drawn through a water bath (multiple water streams) at a temperature of 85 °C controlled by the hot plate to promote the polymer chain alignment and to remove the DMF solvent, and the fibers were soaked in methanol for an additional time of 24 h to further coagulate the fiber. The wet PAN fiber was dried at a temperature of 50 °C under a vacuum in the oven, the moisture in the fibers was removed and the voids collapsed, later the fibers were drawn in an oil bath (an oil-in-water emulsion) with varying temperatures of 125, 135, and 145 °C, consecutively, for a maximized molecular extension and to add a protective layer on the fibers to avoid the defects, the highest draw ratio fibers were collected on fiber winders (Figure 1e) at 145 °C. To promote the orientation of GF, three-phase fibers were drawn at these different temperatures so that the layered fiber structure encountered higher and higher temperatures, overcoming the barriers of GF rotation momentum and GF had more preferentially aligned morphologies along the fiber axis. PAN fibers were stretched at high temperatures to increase the degree of molecular orientation and to eliminate internal stress, resulting in a dense structure and optimized mechanical properties.

#### 2.2.5 Fiber Annealing

During the postprocessing of spun fibers, residual stresses from the hot drawing stage leading to microstructural imperfections and unstable/metastable conformational chain states can be eliminated to enhance the overall fiber quality and performance. All the spinnable fibers (Table 1) were heat treated in an oxidative atmosphere in the tube furnace (Figure 1f) with a temperature of 250–350 °C at different heating rates for 1.5 h and later cooled to room temperature at a rate of 1 °C/min to produce stabilized fibers (Figure 1g).

### 2.3 Characterizations

Single fiber uniaxial tests were conducted using a tensile tester (Discovery HR-2 hybrid rheometer, TA Instruments Inc., USA) (see some of the mechanical test curves in the Supporting Information, Figure S2). A gauge length of 20 cm was used with a constant linear strain rate of 50 μm/s for fibers drawn at different stages. A number of 5–7 samples of each fiber type were tested to obtain the mechanical parameters, including Young’s modulus, tensile strength, tensile strain, and toughness. Differential scanning calorimetry (DSC) (DSC 250, TA Instruments Inc., USA) was conducted on 2 mg fiber samples for each fiber type, and the temperature increased from room temperature (RT) (∼25 °C) to 370 °C with a different heating rate of 5–25 °C/min in a nitrogen atmosphere to understand the cyclization behaviors, followed by reruns in the air for oxidation and cross-linking studies. The filler (i.e., GF) dispersion in the polymer (i.e., PAN) and the fiber morphological features of different PAN/GF concentrations were observed using scanning electron microscopy (SEM) (i.e., SEM/focused ion beam (FIB), Auriga (Zeiss)) at an operating voltage of 5 kV. All of the fibers were mounted on a 90° cross-sectional stub with a fractured surface facing up and were coated with a thin gold layer (15–20 nm) before SEM imaging.

## 3 Results and Discussion

### 3.1 Fiber Spinning Method Rationale

Our unique spinning technique, primarily used for the 12 wt % PAN and the 12 wt % PAN/GF composite fibers (Figure 1), was based on dry-jet wet-spinning. We employed an in-house-developed coaxial spinneret to create a three-layered fiber. The process started with the extrusion of spinning dope through a spinneret (Figure 1b), with flow rates precisely controlled by syringe pumps (Figure 1a). The emerging fibers passed through a methanol coagulation bath (Figure 1c) at room temperature (approximately 25 °C) before being collected on winders. The fibers consistently decreased in diameter after specific hot drawing stages (Figure 1d). This high shear force aligned macromolecules in a more parallel manner along the fiber axis. Subsequently, the fibers underwent drawing in an oil bath (an oil-in-water emulsion) at varying temperatures of 125, 135, and 145 °C, successively, to maximize molecular extension without oxidation and degradation, thus achieving the highest draw ratio (Table S1). The fibers were then collected on fiber winders (Figure 1e). Postdrawing procedures were carefully controlled with programmable heating rates and temperatures via a tube furnace (Figure 1f), before assessing the mechanical, thermal, and morphological properties of these PAN and three-phase composite fibers (Figure 1g). Throughout the drawing and post-treatment procedures, the exterior PAN layer (indicated in purple) was expected to form more extended polymer chains than the inner PAN layer (also indicated in purple), generating shear stress on the middle layer consisting of GF (indicated in red) (Figure 1h). Consequently, this shear stress applied during the fiber thinning process compelled better GF alignment along the fiber axis than simply mixed compositions without a layered microstructure, resulting in higher mechanical reinforcement.^39^

Table 1 lists the fiber processability, testability, and drawing parameters. All samples were spinnable, with stable quality and minimized defect density after optimizing the fabrication parameters (e.g., injection rates, air gap, flow rates in coagulant, and collection rates) (Table 1). The addition of GFs and their effects on the fiber stretchability and dimensions were compared with the pure PAN. The draw ratio increased with proper GF additions (e.g., 0.1, 1.0, and 2.5 wt %) as compared to the pure PAN. The direct contact of the outer layer of the fiber with the hot medium led to more macromolecular stretching than the inner layer due to the heat transfer efficiency, with the GF facilitating the extension of polymer chains due to the lubrication effects in the middle layer.^40^ As a result, the stacked particles underwent stepwise exfoliation and aligned in the axial direction (Table S2). The efficiency of exfoliation and alignment in the three-phase fibers relied on the initial thickness of the channel and could be controlled by adjusting the spinneret outlet dimension and air gap distance.^41^ However, higher GF inclusions in the fiber middle layer (i.e., >2.5 wt %, as shown in Table 1) resulted in decreased drawability compared to pure PAN fibers. For example, the presence of 200 wt % GF in 12 and 10 wt % PAN fibers showed a decreased total draw ratio of 28 and 20, with the final fiber diameter of 224 and 156 μm, respectively, both much larger than the pure PAN fibers (i.e., DR of 32.5 and diameter of 83 μm).

### 3.2 Mechanical Analysis

#### 3.2.1 Effect of Fiber Drawability on Mechanical Properties

**Figure 2:**
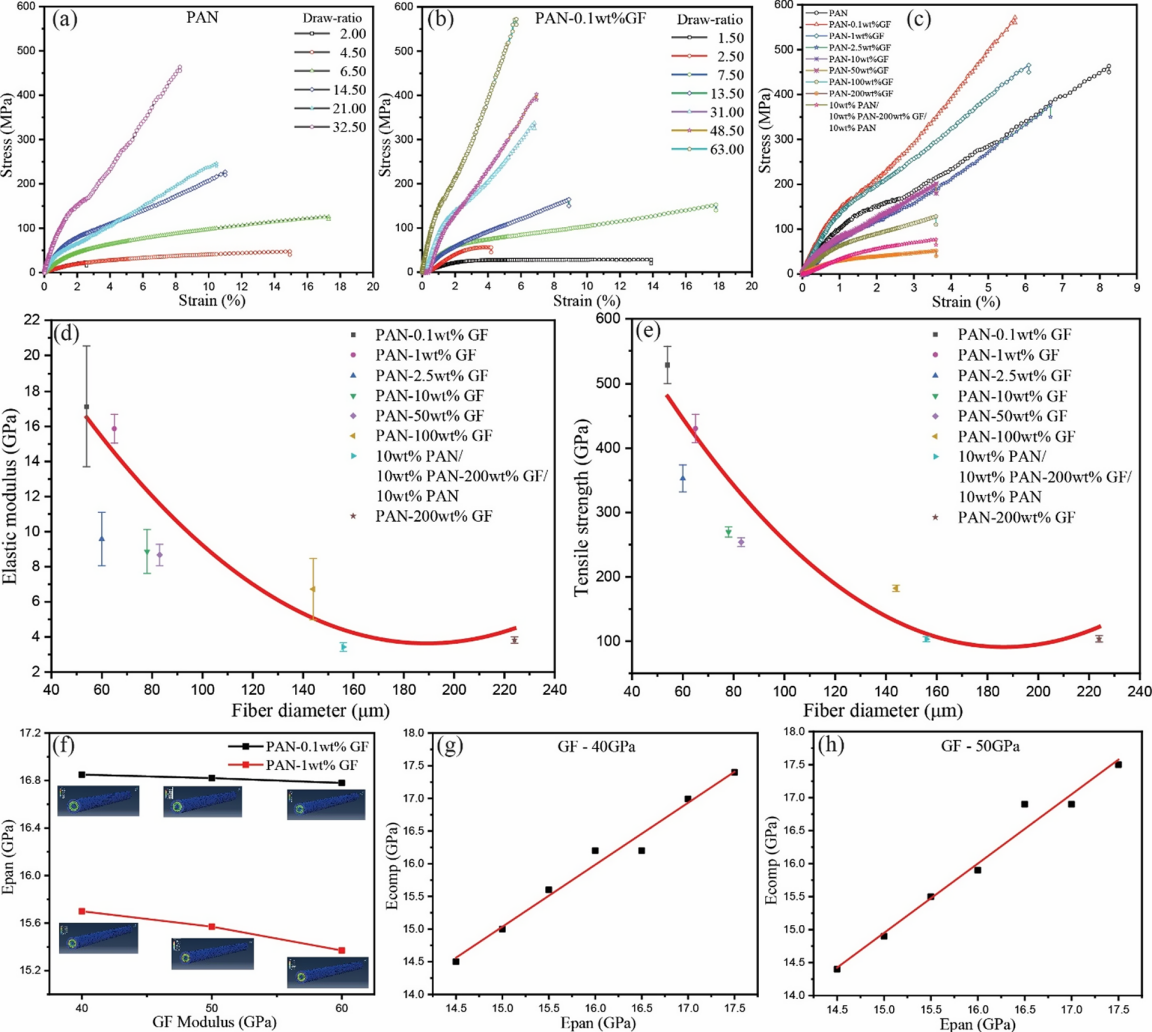
Mechanical properties of drawn fibers with (a) PAN fibers showing increasing mechanical properties (e.g., strength, modulus) as a function of the increasing draw ratio, (b) PAN-0.1 wt % GF fibers showing increasing mechanical properties with increasing draw ratios, and (c) effect of increasing GF concentrations on the mechanical properties showing the highest increase in 0.1 wt % GF addition as compared with pure PAN after the drawing procedures. The scale/size effects show the average (d) elastic modulus and (e) tensile strength values as a function of the fiber diameter. Simulation results show that (f) Young’s modulus of bulk PAN in the composites is higher than the 14.55 GPa in pure PAN fibers for both 0.1 and 1 wt % GF addition, suggesting the influence of GF on PAN morphologies and microstructures. To understand the reinforcement mechanism, we conducted modeling and simulation via the finite element method (FEM) using ABAQUS. (f) Mechanical contribution from PAN in the composite was calculated based on the tested composite properties (Table 2), glass fiber and PAN percentage (experimental design, see Table 2), and parametric studies of GF modulus (i.e., 40, 50, and 60 GPa based on literature research). For example, tuning the bulk PAN’s Young’s modulus between 14.5 and 17.5 GPa, (g) with a GF modulus of 40 GPa, and similarly, (h) with a GF modulus of 50 GPa, would generate the composite modulus. Based on the simulated results, the fitted equation will generate an accurate PAN modulus matching the composite stiffness at a specific GF percentage (i.e., 17.12 GPa for 0.1 wt % GF composites and 15.87 GPa for 1 wt % GF composites). See Supporting Information Figure S4 for more details.

Fiber drawability serves as an essential metric not only indicating their processability (as presented in Table 1) but also reflecting the extent of polymer chain alignment and subsequent mechanical property developments, as illustrated in Figure 2. The stress–strain curves, available in Figure 2, and the corresponding mechanical property data listed in Table 2 provide valuable insights. Figure 2a demonstrates that PAN fibers consistently displayed an increase in Young’s modulus and ultimate tensile strength values as the DR increased. In the initial phases of the drawing process, the fibers may display notable porosity and voids.^42^ These characteristics can lead to the formation of defects and fractures, even when subjected to relatively low levels of stress. Consequently, this can lead to a decrease in the mechanical performance and strength of the fibers (low elongation break at the first few drawing stages, as shown in Table S1). As the drawing process advances, with the fibers experiencing a higher draw ratio, their strength typically shows an increase. At a final stage draw ratio of 32.5 for 12 wt % PAN, the modulus reached 14.55 ± 1.82 GPa, with a tensile strength of 460.00 ± 15.00 MPa (Figure S2). Notably, these enhancements were substantial, with the modulus being 16.16 times higher and the tensile strength being 23.47 times higher compared to fibers with a draw ratio of 2.00 (Table S3). As compared, in Figure 2b, the three-phase layered fiber exhibited a similar trend of increasing modulus with the highest value reaching 17.12 ± 3.42 GPa and tensile strength of 572.25 ± 31.00 MPa as the DR increased. It is worth mentioning that further improvements in mechanical properties can be achieved by increasing the draw ratios but will have to sacrifice the windability (e.g., larger draw ratios for random fiber segments and shorter fiber collections). Importantly, all fibers produced in this study exhibited excellent processability, allowing for continuous collection on winders, surpassing 120 m lengths.

**Table 2:** Summary of Fiber Drawing and Mechanical Properties of Fibers

fiber type	mechanical properties of prestabilized fibers		
	Young's modulus (GPa)	tensile strength (MPa)	elongation at break (%)
PAN	14.55 ± 1.82	460.00 ± 15.00	7.40 ± 1.57
*PAN-0.1 wt % GF*	*17.12 ± 3.42*	*572.25 ± 31.00*	*5.72 ± 0.06*
*PAN-1.0 wt % GF*	*15.87 ± 0.82*	*465.20 ± 24.00*	*6.09 ± 0.72*
PAN-2.5 wt % GF	9.58 ± 1.52	380.50 ± 23.00	6.73 ± 1.18
PAN-10 wt % GF	8.87 ± 1.25	289.75 ± 8.50	5.41 ± 0.46
PAN-50 wt % GF	8.67 ± 0.61	272.50 ± 7.50	4.97 ± 1.37
PAN-100 wt % GF	6.72 ± 1.75	194.30 ± 5.40	6.08 ± 0.20
PAN-200 wt % GF	3.82 ± 0.25	108.56 ± 5.45	10.86 ± 1.18
10 wt % PAN/10 wt % PAN-200 wt % GF/10 wt % PAN	3.42 ± 0.18	108.10 ± 4.50	7.76 ± 0.26

The addition of GF waste had a notable impact on fiber drawability, as indicated in Table 1, subsequently influencing the mechanical properties of the resulting composites (Figure 2c and Table 2). As depicted in Figure 2c, even with a minimal GF concentration of 0.1 wt %, the composite fibers exhibited a significant improvement. They displayed a modulus of 17.12 ± 3.42 GPa and a tensile strength of 572.25 ± 31.00 MPa compared to pure PAN fibers, which had a modulus of 14.55 ± 1.82 GPa and a tensile strength of 460.00 ± 15.00 MPa. However, a further increase in GF weight percentage, from 0.1 to 200 wt % within the middle layer of the fiber, resulted in a consistent decrease in both Young’s modulus (i.e., with an average modulus decreasing from 17.12 to 3.82 GPa) and ultimate tensile strength (i.e., with an average strength dropping from 572.25 to 108.56 MPa) of the composite fibers. This suggests the potential agglomeration of GF waste and its hindrance to high molecular chain extension (Figure 2c).

#### 3.2.2 Effect of GF Loading Percentage on the Fiber Size and Defect Density

As previously discussed, the mechanical properties are significantly affected by the loading of glass fibers, as it involves a delicate balance between GF reinforcement and the defect density induced by GF agglomeration (Table 2). A higher GF content generally leads to enhanced mechanical properties, assuming that the GF is well-dispersed and interacts efficiently with the matrix. However, in our middle layer with a high GF content, achieving uniform distribution can be challenging. This nonuniformity can result in friction-based sliding and crack initiation between glass fibers, ultimately reducing the mechanical integrity of the composite fiber.^43^ This challenge becomes more pronounced at extreme filler loadings, such as 200 wt % GF, as seen in this study. The irregular dispersion and distribution of fillers play a critical role in determining the composite fiber’s performance. The lower strength observed in the composite samples (Table 2) can be attributed to several factors. One of these factors is the presence of residual resin debris and GF segments with ineffective lengths in critical areas, increasing the likelihood of microcrack formation. Moreover, the uneven distribution of resin particles from the recycled turbine blades on the GF surface, particularly at the GF ends, contributes to a decrease in the ultimate strength of the composite samples. Additionally, composite samples with a higher GF/PAN content tend to exhibit a greater number of pores, which can compromise the overall structural integrity and reduce material strength. This is evident when comparing the 12 wt % PAN-based sample (i.e., PAN-200 wt % GF) with the 10 wt % PAN-based samples (i.e., 10 wt % PAN/10 wt % PAN-200 wt %GF/10 wt % PAN) with a more detailed fiber structure in Figure S6.

Reducing the direct interaction between GF and increasing the contact area between GF and the polymer matrix can be achieved by decreasing the fiber diameter, as evident in the 0.1 wt % GF samples with a diameter of 54 μm (Table 1). Ideally, this fiber size reduction results in the middle layer having a single GF thickness. During the fiber drawing process, GF bundles undergo extension and reorganization, aligning preferentially along the composite fiber axis (Figure 1). Notably, a clear correlation between a smaller diameter and higher mechanical properties was observed (Figure 2d,e). This observation aligns with the principles of classic fracture mechanics, with the GF concentration significantly influencing the fiber size. According to Griffith’s theory, smaller diameter fibers, as initially demonstrated in glass fibers, tend to approach theoretical predictions for tenacity and strength as defects decrease.^44,45^ Decreasing the diameter effectively reduces defect density, nonlinearly dependent on the fiber loadings, allowing fiber properties to approach their theoretical limits.^46^

#### 3.2.3 Effect of Low GF Loading on Macromolecular Behaviors from Mechanics Perspectives

Recycled materials are often employed as fillers or reinforcements in industries where the incorporation level of the reinforcement or filler is limited to under 10 wt % due to the poorer quality of recycled solids than virgin materials. These lower-quality dispersions often contain agglomerations or entanglements that not only initiate cracks but also hinder polymer crystallization, impeding effective alignment.^47^ Working with low-reinforcement particle loadings, such as glass fibers (GF), can be advantageous. Low filler additions can promote interfacial crystal growth and facilitate unique crystal structures, enabling better polymer/particle interactions and precise control over the properties of the polymer composite.^48^ Notably, Table 2 demonstrates that incorporating 0.1 wt % GF in the fiber middle layer results in significant improvements in the fiber’s overall mechanical properties. Compared with pure PAN fibers, the composite fiber exhibits a remarkable 24.4% increase in tensile strength and a notable 17.66% increase in tensile modulus. However, these enhancements cannot be explained by conventional composite mechanics like the rule of mixture.
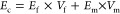
1Here, *E* represents the modulus, c is the composite, f is the filler, m is the matrix, and *V* is the volume fraction. In this case, a 0.1 wt % GF translates to only 0.017 vol % within the entire PAN matrix, and considering the ideal GF modulus of 40–60 GPa, its contribution is negligibly low, amounting to less than 0.01 GPa. Consequently, the influences of the low-concentration GF on the PAN microstructure and performance warrant further investigation. It is worth noting that the standard deviations for lower wt % GF fibers were high, and the mechanical data were more scattered than those fibers without defects. High standard deviation in their strength indicated that the presence of defects, such as porosity and voids, within the gauge length, can significantly affect the mechanical properties of the fibers.

The Weibull analysis, a statistical technique, evaluates the strength distribution and failure probabilities of individual fibers. It assesses variability, predicts failure likelihood at different stress levels, aids in reliability assessment, and informs material selection and design for enhanced performance and durability in composite materials.^49^ In this study, we consider the final DR fibers for the Weibull analysis of both PAN and PAN-0.1 wt % GF fibers (Figure S3) for the tensile tests. The Weibull distribution here enabled the assessment of our fiber strength distributions and prediction of failure probabilities under various stress conditions. It is essential for comparing materials and ensuring the reliability of engineered systems. Equations S1 and S2 provide the cumulative distribution function for the two-parameter Weibull distribution, where β represents fiber stability, *x*_o_ means the Weibull strength and the equations are widely used to fit experimental data. Comprehending the significance of these fitting parameters aids in the assessment of the material integrity through the probability of failure. As demonstrated in Table S4, the composite fibers exhibit higher Weibull strength, aligning with the fracture strength observed in tensile tests. Simultaneously, a similar fitting can be applied to the Weibull modulus. In contrast to the Weibull strength, the Weibull modulus is more influenced by factors related to reinforcement fillers, such as the intrinsic properties and alignment effects of glass fibers (GF). GF alignment affects the modulus, while both strength and failure probability are determined by the weakest point. Following the rule-of-mixture^50^ series model, even slight misorientation in the PAN/GF layer significantly impacts the modulus, resulting in a drop in modulus.

**Figure 3:**
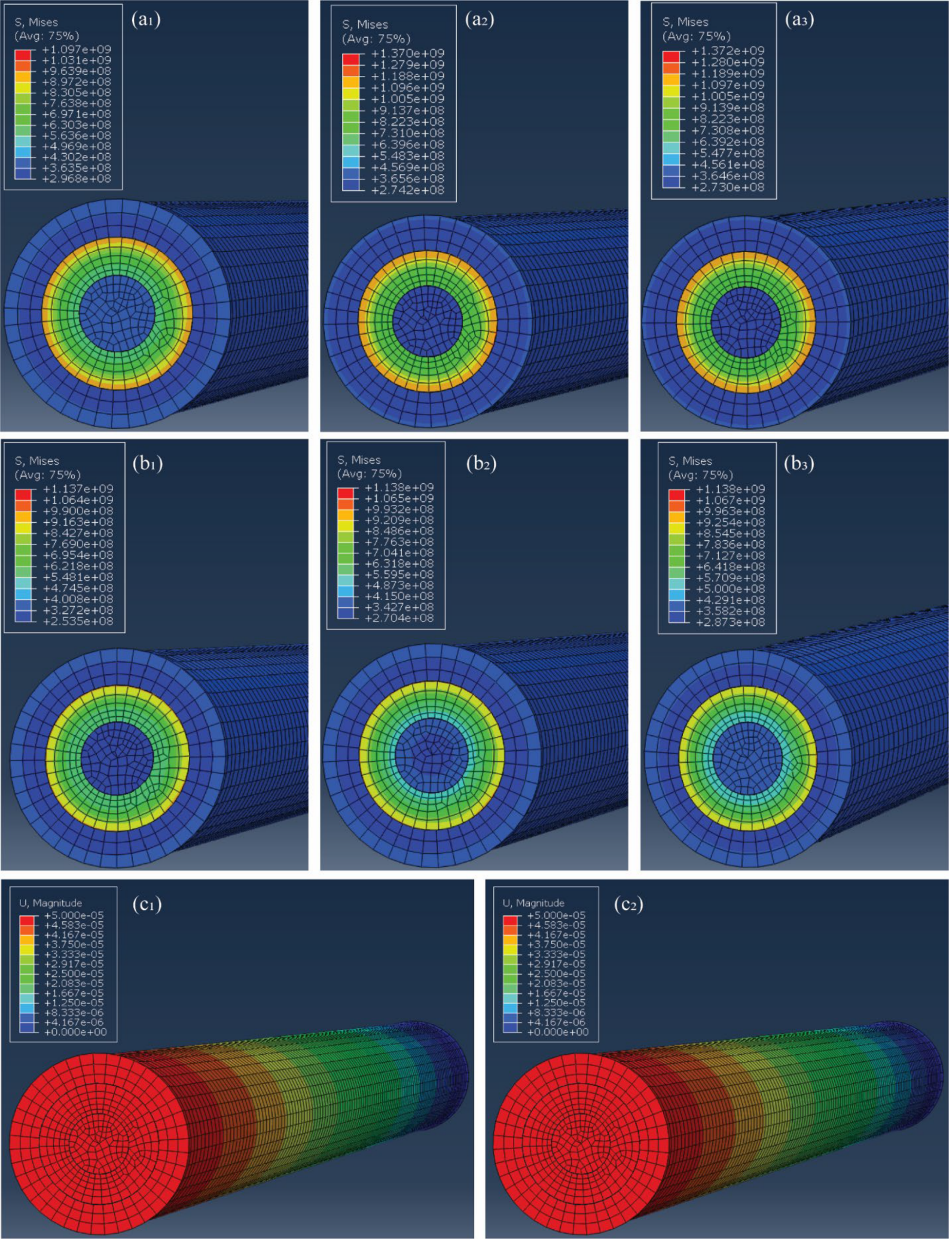
Computational depiction of stress variation in the three-layered composite using FEA through ABAQUS software for 0.1 wt % GF with varying GF modulus of 40, 50, and 60 GPa, respectively in (a_1_), (a_2_), and (a_3_) and for 1 wt % GF with varying GF modulus of 40, 50, and 60 GPa, respectively, as seen in (b_1_), (b_2_), and (b_3_). The displacement contours for the fibers at a constant strain rate of 50 μm/min for 0.1 wt % GF (c_1_) and 1 wt % GF (c_2_).

To assess how glass fibers (GF) influence the modulus of PAN, we utilized the finite element method (FEM) (via the ABAQUS software) for visualizing the elastic behavior within the PAN/GF composite, as depicted in Figures 2f and 3. The simulation validated the conditions of a uniaxial tensile test performed on the PAN/GF composite. Various parameters were taken into account, including the fiber volume fraction, the pure PAN elastic modulus (ranging from 14.5 to 17.5 GPa), and the GF modulus (ranging 40–60 GPa^51,52^). The goal was to determine the overall longitudinal modulus of the composite. Subsequently, to determine the PAN elastic modulus in the 0.1 and 1 wt % GF composites after reinforcement and composite integration, a range of modulus values was acquired. These values were then interpolated with experimental values of 17.12 and 15.87 GPa.

**Figure 4:**
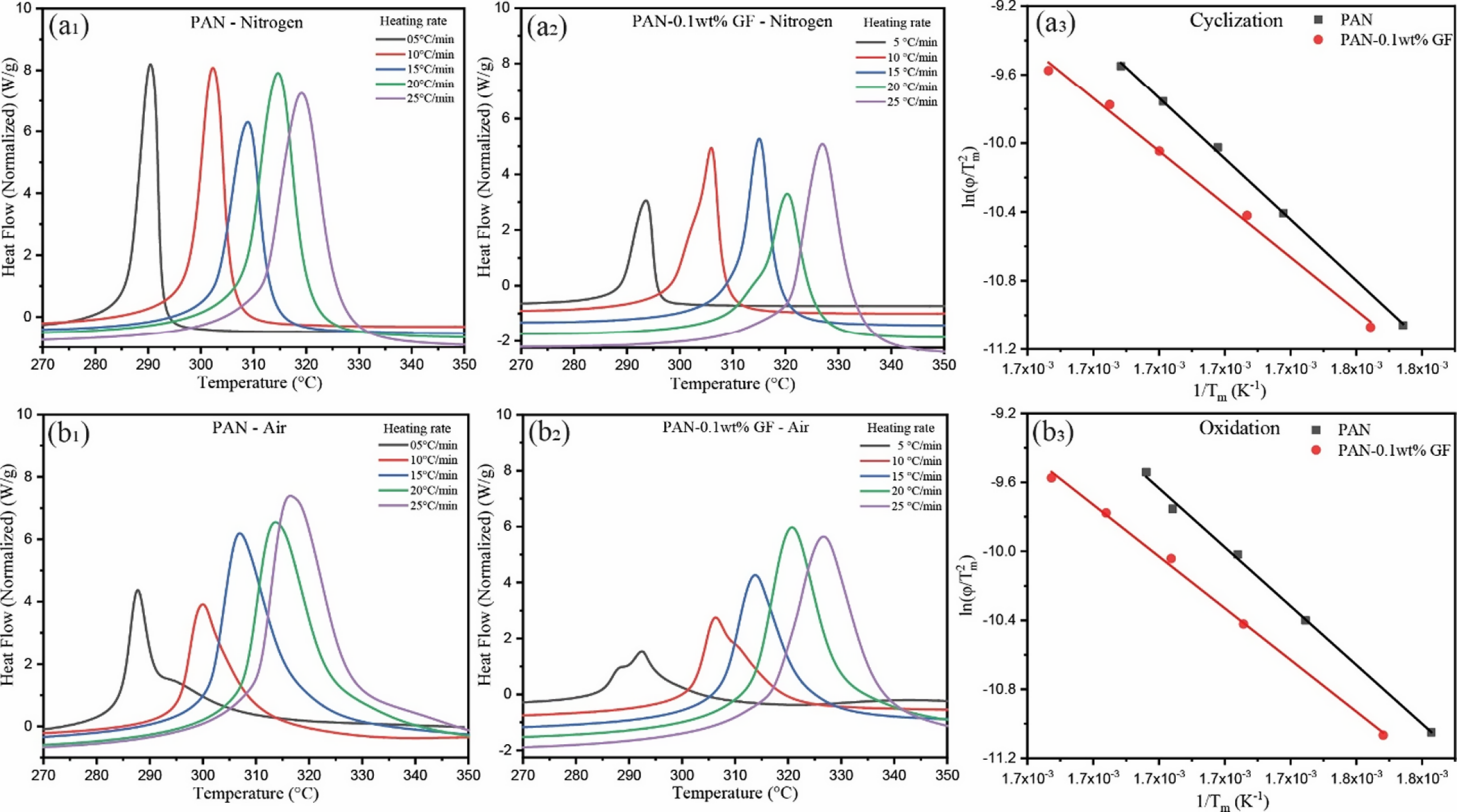
DSC curves in the nitrogen atmosphere with different heating rates for (a_1_) PAN and (a_2_) PAN-0.1 wt % GF, followed by the DSC curves in the air with different heating rates for (b_1_) PAN and (b_2_) PAN-0.1 wt % GF. Plots of ln (φ/*T*_m_^2^) versus 1/*T*_m_ according to the Kissinger method for PAN and PAN/GF fibers, showing the (a_3_) cyclization and (b_3_) oxidation reaction kinetics. See Supporting Information Table S7 for activation energies for different fiber types.

**Figure 5:**
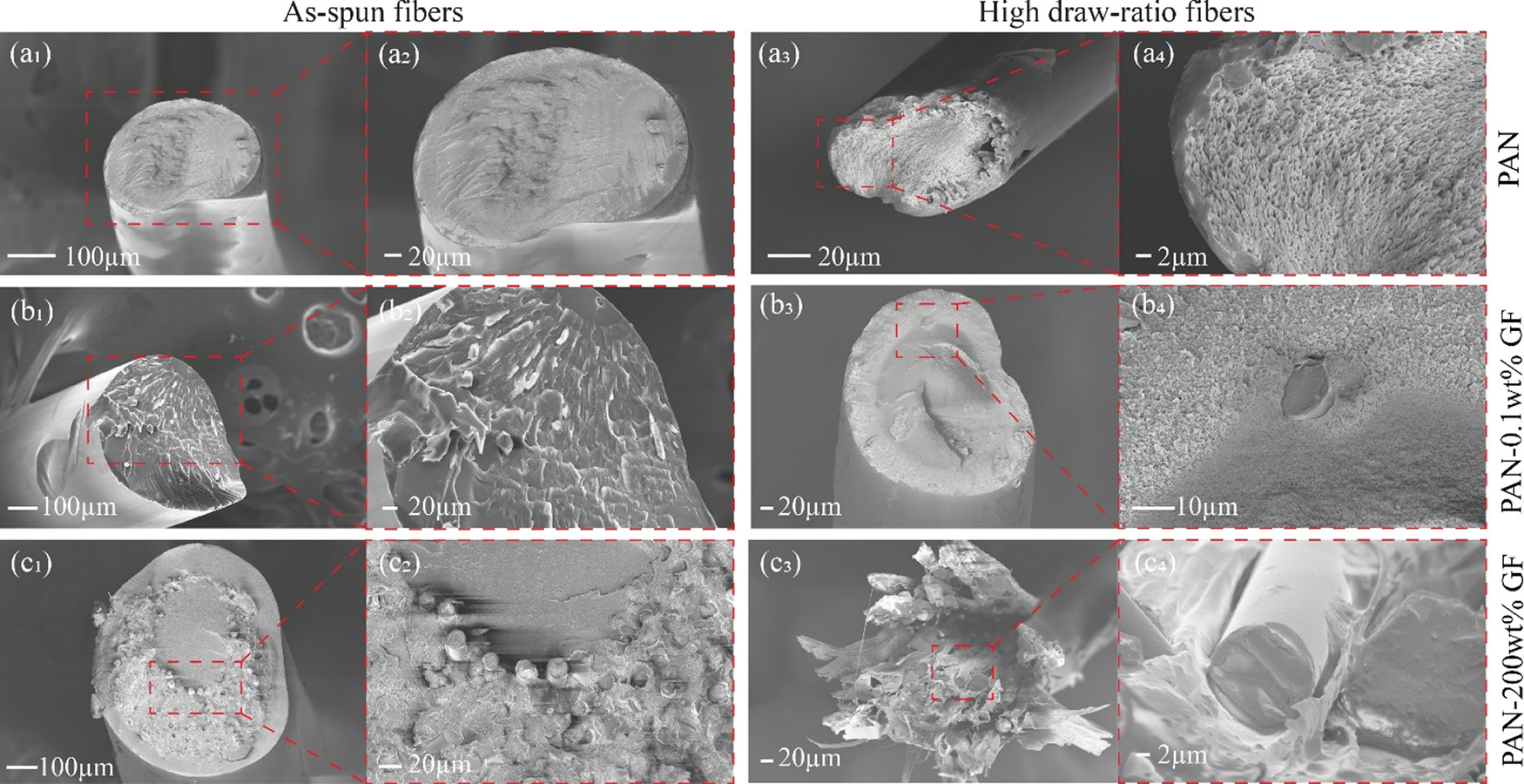
Cross-sectional SEM images of as-spun and highly drawn fibers for (a_1_–a_4_) PAN, (b_1_–b_4_) PAN-0.1 wt % GF, and (c_1_**–**c_4_) PAN-200 wt % GF samples. See Supporting Information Figures S6–S8 for different wt % GF morphologies.

The FEM was employed to perform a micromechanical elastic analysis of a unidirectional PAN/GF composite (Figure 3). This analysis involved several key assumptions in the modeling process, including the uniformity of elastic moduli and tensile strength within the fibers along the Representative Volume Element (RVE), homogeneity in the composite layer where glass fibers were experimentally reinforced, the absence of atomic-level polymer inclusions (void-free composite), and the use of a scaled-up geometry in the contour representation to maintain mesh linearity and reduce potential distortions. A uniaxial tensile test configuration was replicated, applying strain load exclusively in the *z*-direction while constraining the rare and front ends in all other directions. By fitting the composite modulus to the experimental measurements (refer to Table 2), we were able to determine the intrinsic modulus of PAN (as illustrated in Figure 2g,h for 40 and 50 GPa and Figure S4 for 60 GPa, respectively). The analysis indicates that PAN’s contribution to the mechanical properties in the composites exceeds that in pure PAN (Table 3), suggesting that the presence of glass fibers significantly influences PAN morphologies and microstructures. This influence could be attributed to lubrication during fiber spinning and drawing, which requires an understanding of their microstructures and thermal behavior (see Figure 4). Establishing a quantitative relationship between these variables is crucial for effective composite design. This relationship is further supported by comparing SEM images (see Figure 5) of actual samples and simulated models’ RVEs (as simulated in Figure 3). The presence of GF clusters introduces microstructural variation, leading to variable stress distribution and elastic strain across the composite RVE. Additionally, an increase in fiber concentration results in the observation of tangled and misoriented fibers, causing a decrease in the elastic modulus. Notably, the influence of the fiber volume content consistently appears in both experimental and computational scenarios. An important observation is the inverse relationship between GF volume and the composite Young’s modulus. This relationship displays a linear trend, indicating a consistent decrease in the elastic modulus with increasing fiber volume. Furthermore, in regions with concentrated agglomerates, stress transmission to individual fibers may be less efficient, potentially causing stress concentrations and an overall reduction in the composite stiffness.

**Table 3:** Comparing Young’s Modulus of PAN in the Composites to the Pure PAN Fibers per the Simulated Values

PAN modulus in control fibers (GPa) from experiments	PAN modulus in PAN-0.1 wt % GF (GPa) derived from simulations	PAN modulus in PAN-1 wt % GF (GPa) derived from simulations	middle layer GF modulus (GPa)
14.55	16.85	15.70	40
	16.82	15.57	50
	16.78	15.37	60

### 3.3 Kinetic Analysis of Fibers

The thermal stabilization process induces both chemical and physical changes in PAN-based fibers (Table 4). To study these changes, we employed thermal analysis, specifically DSC, to record the heat flow during thermal transitions in the polymer. DSC provides essential data, such as peak temperatures and associated enthalpy changes. To further understand the polymer’s behavior, we utilized the Kissinger method, which calculates the activation energy based on the peak temperatures obtained from the DSC results. Activation energy is the energy required for specific thermal processes. The activation energy values were obtained using the Kissinger equation.
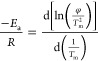
2where *E*_a_ is the activation energy (kJ/mol), φ is the heating rate (°C/min), *R* is the molar gas constant, and *T*_m_ is the peak temperature (K), *E*_a_ was taken as the slope of the plots.

**Table 4:** DSC Data for PAN and PAN-0.1 wt % GF Fibers

heating rate (°C/min)/peak temperature (*T*_m_)	in air		in nitrogen	
	PAN (°C)	PAN-0.1 wt % GF (°C)	PAN (°C)	PAN-0.1 wt % GF (°C)
5	287.76	292.41	290.47	293.63
10	300.06	306.33	302.28	305.96
15	306.91	313.80	308.94	315.05
20	313.66	320.70	314.67	320.32
25	316.43	326.62	319.13	326.96

When subjecting the fibers to increasing temperatures (up to 350 °C) with a corresponding rise in the heating rate under a nitrogen atmosphere, highly crystalline fibers typically display a sharp exothermic peak at a specific temperature. This peak is attributed to the cyclization reaction (as observed in Figure 4a_1_ for PAN and Figure 4a_2_ for PAN-0.1 wt % GF). However, in an air atmosphere, the peak broadens due to the simultaneous occurrence of complex chemical reactions. At a heating rate of 5 °C/min, a second peak appears, reflecting the intricate interplay of cross-linking and oxidation reactions (Figure 4b_1_ for PAN and Figure 4b_2_ for PAN-0.1 wt % GF). We further investigated the kinetics and activation energies of these processes by subjecting the highest draw fibers to different heating rates (ranging from 5 to 25 °C/min). Increasing the heating rate led to a shift in the peak exotherm to higher temperatures in both the nitrogen and air atmospheres. This shift is attributed to the reduced time available for the fibers to complete their reactions, resulting from increased thermal effects and thermal inertia. Notably, the addition of GF influences the thermal behavior and elevates the peak to higher temperatures, as summarized in Table 4. We also exposed different fiber types to a progressively increasing temperature, reaching up to 350 °C in both air and nitrogen environments and recorded the peak temperatures (see Tables S5 and S6). It is worth noting that a shift in the peak positions toward higher temperatures was observed, particularly in the nitrogen atmosphere, as depicted in Figure S5.

As indicated in Table 5, it is evident that the PAN-0.1 wt % GF composite fiber displays a notably higher rate constant for the cyclization reaction compared to pure PAN fiber. This observation underscores the profound impact of additional components, such as the GF reinforcing materials, on the cyclization behavior within the composite. The activation energies are observed to decrease in the PAN-0.1 wt % GF composite fibers in comparison to pure PAN fibers, both for cyclization (Figure 4a_3_) and oxidation (Figure 4b_3_). Several factors contribute to this intriguing phenomenon. One plausible explanation might be the interactions that occur between the polymer matrix and the reinforcing GF materials. These interactions can potentially affect the chemical processes within the composite, leading to alterations in the activation energies of these reactions. Moreover, changes in the thermal and mechanical properties of the composite due to the presence of GF could contribute to these observed alterations. Additionally, the interface between the polymer matrix and the GF materials might be promoting enhanced chemical reactivity, which could be another factor influencing the activation energies.^53,54^ Furthermore, the notable increase in the cyclization rate constant within the composite fiber suggests that there is an improvement in the efficiency of the cyclization process or a higher degree of cyclization. This improvement is of significant interest as it can contribute to the formation of a more stable and ordered polymer structure, which is a crucial factor for enhancing the overall performance and properties of the composite material.^55−57^

**Table 5:** Activation Energies (kJ/mol) Were Determined from the Kissinger Method for PAN and PAN-0.1 wt % GF

chemical reaction	PAN (kJ/mol)	PAN-0.1 wt % GF (kJ/mol)
oxidation	157.02	120.70
cyclization	144.20	124.70

### 3.4 Fiber Morphologies via SEM Characterizations

The fractured surfaces of both pure PAN (Figure 5a_1_–a_4_) and the composites exhibit notable differences in surface morphologies, particularly in terms of the dispersion patterns of the GF fibers. The degree of alignment, as visually apparent in the SEM images, offers qualitative insight into the orientation of the fibers. This orientation is a pivotal factor influencing the overall mechanical behavior and anisotropic properties of the composite. An important observation is that highly drawn fibers consistently display significantly smaller diameters than their as-spun counterparts, regardless of fiber composition, which is depicted in Figure 5. This aligns with the data provided in Table 1. Both PAN-0.1 wt % GF and PAN-200 wt % GF composites feature a three-layered fiber structure, with the middle layer containing varying amounts of GF (Figure 5b,c). PAN-0.1 wt % GF exhibits an even distribution of GFs in the middle layer, while PAN-200 wt % GF (Figures S6 and S7 with different PAN content) displays heavily agglomerated GF groups. It is worth noting that all of the GF particles are observed protruding through the cross-section, which significantly contributes to the mechanical reinforcement along the fiber direction. In cases where the GF are homogeneously dispersed and preferentially aligned along the fiber axis (Figure 5b_1_,b_2_), the reinforcement efficiency is notably higher. This results in improvements in the tensile strength, modulus, and various other mechanical properties, as outlined in Table 2. Highly loaded GFs were expected to provide additional strength and stiffness, further enhancing these properties. However, excessive agglomeration, especially with an increasing GF loading (Figure 5c_1_,c_2_), creates stress concentration points. This renders the fibers more prone to failure under mechanical load. Moreover, the agglomerates hinder effective dispersion and interfacial bonding between the particles and the polymer matrix, thereby limiting the desired enhancements in material properties. Notably, the broken fiber ends protruding from the fracture surface (Figure 5c_3_,c_4_) indicate that the fibers were longitudinally aligned in the direction of the applied force. Due to the interference of the GF with the PAN matrix, the fibers ruptured and were subsequently pulled out. This comprehensive analysis of the SEM images sheds light on the intricate relationship between fiber alignment, composite composition, and resulting mechanical properties, offering a deeper understanding of the material’s behavior under mechanical stress. All of the spinnable fibers underwent oxidative heat treatment in a tube furnace with variable heating rates and were gradually cooled to room temperature to produce stabilized fibers (Figure S9).

## 4 Conclusions

In conclusion, our study underscores the significant potential of mechanical recycling in repurposing composite wind turbine blades and recovering valuable glass fibers. Irrespective of GF concentration, a focus on smaller diameter fibers emerges as a promising avenue for improving composite material performance by reducing defects and enhancing mechanical properties. The correlation between fiber diameter and experimental modulus and strength is evident in our data, with smaller-diameter fibers consistently demonstrating superior performance. During the spinning process, the exterior PAN layer of the fiber, in the proximity of the hot plate, facilitated polymer chain stretching above the glass transition temperature. This resulted in shear stress, gradually aligning the glass fibers along the fiber axis, thereby bolstering the mechanical properties of the composite. The notable enhancement in reinforcement efficiency between one-phase and three-layered fibers can be attributed to the layered structure achieved using a 3D printed spinneret in the fiber spinning process. The PAN/GF coaxial-layered fibers we manufactured exhibited remarkable mechanical properties and a lightweight nature, surpassing pure PAN polymer. This advancement offers a sustainable solution for composite material development with substantial increases in both strength and modulus. Such fibers hold immense promise for various industrial applications, notably in enhancing electric vehicles such as body panels and chassis components and other sustainable solutions toward a more environmentally conscious future.

## Supporting Information Available

Additional information includes details related to wind turbine blade recycling and their mechanical recycling with optical images of glass fibers along with resin and additives; TGA for glass fiber reinforced plastics; fiber drawability as a function of drawing conditions; effect of nanoparticles and their influence on polymer crystallization behavior; fiber drawing and mechanical properties of the different draw ration fibers, Weibull analysis includes statistical distribution for tensile test and Young’s modulus; ABAQUS simulation modeling for theoretical prediction of mechanical properties for three-layered composite fibers; DSC curves for different draw ratio fibers in nitrogen and air; activation energy of PAN and their composites determined from the Kissinger method; X-ray diffraction to evaluate the degree of fiber alignment as a function of the draw ratio; SEM images of different wt % GF and their alignment along the fiber axis; heat treatment of composite fibers; and technoeconomic analysis of the composite fibers (PDF)

## References

[ref1] Borrelle S. B.; Ringma J.; Lavender Law K.; Monnahan C. C.; Lebreton L.; McGivern A.; Murphy E.; Jambeck J.; Leonard G. H.; Hilleary M. A.; Eriksen M.; Possingham H. P.; De Frond H.; Gerber L. R.; Polidoro B.; Tahir A.; Bernard M.; Mallos N.; Barnes M.; Rochman C. M. Predicted Growth in Plastic Waste Exceeds Efforts to Mitigate Plastic Pollution. Science 2020, 369 (6510), 1515–1518. 10.1126/science.aba3656. 32943526

[ref2] Saquing J. M.; Saquing C. D.; Knappe D. R. U.; Barlaz M. A. Impact of Plastics on Fate and Transport of Organic Contaminants in Landfills. Environ. Sci. Technol. 2010, 44 (16), 6396–6402. 10.1021/es101251p. 20704240

[ref3] Zhang Y.; Peng Y.; Peng C.; Wang P.; Lu Y.; He X.; Wang L. Comparison of Detection Methods of Microplastics in Landfill Mineralized Refuse and Selection of Degradation Degree Indexes. Environ. Sci. Technol. 2021, 55 (20), 13802–13811. 10.1021/acs.est.1c02772. 34586798

[ref4] Singh N.; Hui D.; Singh R.; Ahuja I. P. S.; Feo L.; Fraternali F. Recycling of Plastic Solid Waste: A State of Art Review and Future Applications. Compos B Eng. 2017, 115, 409–422. 10.1016/j.compositesb.2016.09.013.

[ref5] Pan D.; Su F.; Liu C.; Guo Z. Research Progress for Plastic Waste Management and Manufacture of Value-Added Products. Adv. Compos Hybrid Mater. 2020, 3 (4), 443–461. 10.1007/s42114-020-00190-0.

[ref6] Jambeck J. R.; Geyer R.; Wilcox C.; Siegler T. R.; Perryman M.; Andrady A.; Narayan R.; Law K. L. Plastic Waste Inputs from Land into the Ocean. Science 2015, 347 (6223), 768–771. 10.1126/science.1260352. 25678662

[ref7] Ross S.; Evans D. The Environmental Effect of Reusing and Recycling a Plastic-Based Packaging System. J. Clean Prod 2003, 11 (5), 561–571. 10.1016/S0959-6526(02)00089-6.

[ref8] Ellis L. D.; Rorrer N. A.; Sullivan K. P.; Otto M.; McGeehan J. E.; Román-Leshkov Y.; Wierckx N.; Beckham G. T. Chemical and Biological Catalysis for Plastics Recycling and Upcycling. Nature Catalysis 2021, 4 (7), 539–556. 10.1038/s41929-021-00648-4.

[ref9] Uekert T.; Singh A.; DesVeaux J. S.; Ghosh T.; Bhatt A.; Yadav G.; Afzal S.; Walzberg J.; Knauer K. M.; Nicholson S. R.; Beckham G. T.; Carpenter A. C. Technical, Economic, and Environmental Comparison of Closed-Loop Recycling Technologies for Common Plastics. ACS Sustain Chem. Eng. 2023, 11 (3), 965–978. 10.1021/acssuschemeng.2c05497.

[ref10] Topham E.; McMillan D.; Bradley S.; Hart E. Recycling Offshore Wind Farms at Decommissioning Stage. Energy Policy 2019, 129, 698–709. 10.1016/j.enpol.2019.01.072.

[ref11] Leon M. J. Recycling of Wind Turbine Blades: Recent Developments. Curr. Opin Green Sustain Chem. 2023, 39, 100746 10.1016/j.cogsc.2022.100746.

[ref12] Beauson J.; Laurent A.; Rudolph D. P.; Pagh Jensen J. The Complex End-of-Life of Wind Turbine Blades: A Review of the European Context. Renewable and Sustainable Energy Reviews 2022, 155, 111847 10.1016/j.rser.2021.111847.

[ref13] Cooperman A.; Eberle A.; Lantz E. Wind Turbine Blade Material in the United States: Quantities, Costs, and End-of-Life Options. Resour Conserv Recycl 2021, 168, 105439 10.1016/j.resconrec.2021.105439.

[ref14] Heng H.; Meng F.; McKechnie J. Wind Turbine Blade Wastes and the Environmental Impacts in Canada. Waste Management 2021, 133, 59–70. 10.1016/j.wasman.2021.07.032. 34385121

[ref15] Pickering S. J. Recycling Technologies for Thermoset Composite Materials—Current Status. Compos Part A Appl. Sci. Manuf 2006, 37 (8), 1206–1215. 10.1016/j.compositesa.2005.05.030.

[ref16] Rani M.; Choudhary P.; Krishnan V.; Zafar S. A Review on Recycling and Reuse Methods for Carbon Fiber/Glass Fiber Composites Waste from Wind Turbine Blades. Composites Part B: Engineering 2021, 215, 108768 10.1016/j.compositesb.2021.108768.

[ref17] Das O.; Babu K.; Shanmugam V.; Sykam K.; Tebyetekerwa M.; Neisiany R. E.; Försth M.; Sas G.; Gonzalez-Libreros J.; Capezza A. J.; Hedenqvist M. S.; Berto F.; Ramakrishna S. Natural and Industrial Wastes for Sustainable and Renewable Polymer Composites. Renewable and Sustainable Energy Reviews 2022, 158, 112054 10.1016/j.rser.2021.112054.

[ref18] Khalid M. Y.; Arif Z. U.; Hossain M.; Umer R. Recycling of Wind Turbine Blades through Modern Recycling Technologies: A Road to Zero Waste. Renewable Energy Focus 2023, 44, 373–389. 10.1016/j.ref.2023.02.001.

[ref19] Li X.; Bai R.; McKechnie J. Environmental and Financial Performance of Mechanical Recycling of Carbon Fibre Reinforced Polymers and Comparison with Conventional Disposal Routes. J. Clean Prod 2016, 127, 451–460. 10.1016/j.jclepro.2016.03.139.

[ref20] Shen M. Y.; Guo Z. H.; Feng W. T. A Study on the Characteristics and Thermal Properties of Modified Regenerated Carbon Fiber Reinforced Thermoplastic Composite Recycled from Waste Wind Turbine Blade Spar. Compos B Eng. 2023, 264, 110878 10.1016/j.compositesb.2023.110878.

[ref21] Zhang J.; Chevali V. S.; Wang H.; Wang C. H. Current Status of Carbon Fibre and Carbon Fibre Composites Recycling. Compos B Eng. 2020, 193, 108053 10.1016/j.compositesb.2020.108053.

[ref22] Palmer J.; Ghita O. R.; Savage L.; Evans K. E. Successful Closed-Loop Recycling of Thermoset Composites. Compos Part A Appl. Sci. Manuf 2009, 40 (4), 490–498. 10.1016/j.compositesa.2009.02.002.

[ref23] Scheirs J. Polymer Recycling: Science, Technology, and Applications; Wiley, 1998, ISBN: 978–0-471–97054–5.

[ref24] Ribeiro M. C. S.; Fiúza A.; Castro A. C. M.; Silva F. G.; Meixedo J. P.; Dinis M. L.; Costa C.; Ferreira F.; Alvim M. R. Recycling of Pultrusion Production Waste into Innovative Concrete-Polymer Composite Solutions. Adv. Mat Res. 2011, 295–297, 561–565. 10.4028/www.scientific.net/AMR.295-297.561.

[ref25] Xu W.; Franklin R.; Ravichandran D.; Bawareth M.; Jambhulkar S.; Zhu Y.; Kakarla M.; Ejaz F.; Kwon B.; Hassan M. K.; Al-Ejji M.; Asadi A.; Chawla N.; Song K. ContinuousNanoparticle Patterning Strategy in Layer-Structured Nanocomposite Fibers. Adv. Funct. Mater. 2022, 32 (35), 2204731 10.1002/adfm.202204731.

[ref26] Xu W.; Ravichandran D.; Jambhulkar S.; Zhu Y.; Song K. Hierarchically Structured Composite Fibers for Real Nanoscale Manipulation of Carbon Nanotubes. Adv. Funct. Mater. 2021, 31 (14), 2009311 10.1002/adfm.202009311.

[ref27] Saleh T. A.; Shetti N. P.; Shanbhag M. M.; Raghava Reddy K.; Aminabhavi T. M. Recent Trends in Functionalized Nanoparticles Loaded Polymeric Composites: An Energy Application. Mater. Sci. Energy Technol. 2020, 3, 515–525. 10.1016/j.mset.2020.05.005.

[ref28] Xu W.; Zhu Y.; Ravichandran D.; Jambhulkar S.; Kakarla M.; Bawareth M.; Lanke S.; Song K. Review of Fiber-Based Three-Dimensional Printing for Applications Ranging from Nanoscale Nanoparticle Alignment to Macroscale Patterning. ACS Applied Nano Materials 2021, 4 (8), 7538–7562. 10.1021/acsanm.1c01408.

[ref29] Cai X.; Riedl B.; Zhang S. Y.; Wan H. The Impact of the Nature of Nanofillers on the Performance of Wood Polymer Nanocomposites. Compos Part A Appl. Sci. Manuf 2008, 39 (5), 727–737. 10.1016/j.compositesa.2008.02.004.

[ref30] Friedrich K.; Zhang Z.; Schlarb A. K. Effects of Various Fillers on the Sliding Wear of Polymer Composites. Compos. Sci. Technol. 2005, 65 (15–16), 2329–2343. 10.1016/j.compscitech.2005.05.028.

[ref31] Ruland W. Carbon Fibers. Adv. Mater. 1990, 2 (11), 528–536. 10.1002/adma.19900021104.

[ref32] Zhang X.; Li B.-W.; Dong L.; Liu H.; Chen W.; Shen Y.; Nan C.-W. Superior Energy Storage Performances of Polymer Nanocomposites via Modification of Filler/Polymer Interfaces. Adv. Mater. Interfaces 2018, 5 (11), 1800096 10.1002/admi.201800096.

[ref33] Yousefi N.; Sun X.; Lin X.; Shen X.; Jia J.; Zhang B.; Tang B.; Chan M.; Kim J.-K.; Yousefi N.; Sun X. Y.; Lin X. Y.; Shen X.; Jia J. J.; Zhang B.; Kim J. K.; Tang B. Z.; Chan M. Highly Aligned Graphene/Polymer Nanocomposites with Excellent Dielectric Properties for High-Performance Electromagnetic Interference Shielding. Adv. Mater. 2014, 26 (31), 5480–5487. 10.1002/adma.201305293. 24715671

[ref34] Yoon J.; Yang H. S.; Lee B. S.; Yu W. R. Recent Progress in Coaxial Electrospinning: New Parameters, Various Structures, and Wide Applications. Adv. Mater. 2018, 30 (42), 1704765 10.1002/adma.201704765. 30152180

[ref35] Chao M.; Wang Y.; Ma D.; Wu X.; Zhang W.; Zhang L.; Wan P. Wearable MXene Nanocomposites-Based Strain Sensor with Tile-like Stacked Hierarchical Microstructure for Broad-Range Ultrasensitive Sensing. Nano Energy 2020, 78, 105187 10.1016/j.nanoen.2020.105187.

[ref36] Yoon S.; Park Y.; Chung Y. S.; Kim S. S.; On S. Y.; Park S. Y.; Kim S. S.; Chung Y. S. Effects of Microwave-Assisted Cross-Linking on the Creep Resistance and Sensing Accuracy of a Coaxial-Structured Fiber Strain Sensor. Adv. Mater. Technol. 2023, 8 (6), 2201615 10.1002/admt.202201615.

[ref37] Liang H.; Lin J.; Jia H.; Chen S.; Qi J.; Cao J.; Lin T.; Fei W.; Feng J. Hierarchical NiCo-LDH@NiOOH Core-Shell Heterostructure on Carbon Fiber Cloth as Battery-like Electrode for Supercapacitor. J. Power Sources 2018, 378, 248–254. 10.1016/j.jpowsour.2017.12.046.

[ref38] Ziabicki A. Introduction. Fundamentals of fibre formation: the science of fibre spinning and drawing; Wiley, 1976, 488.

[ref39] Yamamoto T.; Kanda N. Computational Model for Brownian Dynamics Simulation of Polymer/Clay Nanocomposites under Flow. J. Nonnewton Fluid Mech 2012, 181–182, 1–10. 10.1016/j.jnnfm.2012.06.005.

[ref40] Franklin R.; Xu W.; Ravichandran D.; Jambhulkar S.; Zhu Y.; Song K. Reinforcing Carbonized Polyacrylonitrile Fibers with Nanoscale Graphitic Interface-Layers. J. Mater. Sci. Technol. 2021, 95, 78–87. 10.1016/j.jmst.2021.03.067.

[ref41] Xu W.; Jambhulkar S.; Verma R.; Franklin R.; Ravichandran D.; Song K. In Situ Alignment of Graphene Nanoplatelets in Poly (Vinyl Alcohol) Nanocomposite Fibers with Controlled Stepwise Interfacial Exfoliation. Nanoscale Adv. 2019, 1 (7), 2510–2517. 10.1039/C9NA00191C. 36132729 PMC9417566

[ref42] Liu H. C.; Chien A. T.; Newcomb B. A.; Liu Y.; Kumar S. Processing, Structure, and Properties of Lignin- and CNT-Incorporated Polyacrylonitrile-Based Carbon Fibers. ACS Sustain Chem. Eng. 2015, 3 (9), 1943–1954. 10.1021/acssuschemeng.5b00562.

[ref43] Crosby A. J.; Lee J. Y. Polymer Nanocomposites: The “Nano” Effect on Mechanical Properties. Polym. Rev. 2007, 47 (2), 217–229. 10.1080/15583720701271278.

[ref44] Young K.; Blighe F. M.; Vilatela J. J.; Windle A. H.; Kinloch I. A.; Deng L.; Young R. J.; Coleman J. N. Strong Dependence of Mechanical Properties on Fiber Diameter for Polymer-Nanotube Composite Fibers: Differentiating Defect from Orientation Effects. ACS Nano 2010, 4 (11), 6989–6997. 10.1021/nn102059c. 20945879

[ref45] Nie A.; Bu Y.; Li P.; Zhang Y.; Jin T.; Liu J.; Su Z.; Wang Y.; He J.; Liu Z.; Wang H.; Tian Y.; Yang W. Approaching Diamond’s Theoretical Elasticity and Strength Limits. Nat. Commun. 2019, 10 (1), 5533 10.1038/s41467-019-13378-w. 31797924 PMC6892892

[ref46] Koziol K.; Vilatela J.; Moisala A.; Motta M.; Cunniff P.; Sennett M.; Windle A. High-Performance Carbon Nanotube Fiber. Science 2007, 318 (5858), 1892–1895. 10.1126/science.1147635. 18006708

[ref47] Oliveux G.; Dandy L. O.; Leeke G. A. Current Status of Recycling of Fibre Reinforced Polymers: Review of Technologies, Reuse and Resulting Properties. Prog. Mater. Sci. 2015, 72, 61–99. 10.1016/j.pmatsci.2015.01.004.

[ref48] Fu S. Y.; Feng X. Q.; Lauke B.; Mai Y. W. Effects of Particle Size, Particle/Matrix Interface Adhesion and Particle Loading on Mechanical Properties of Particulate–Polymer Composites. Compos B Eng. 2008, 39 (6), 933–961. 10.1016/j.compositesb.2008.01.002.

[ref49] Weibull W. A Statistical Distribution Function of Wide Applicability. J. Appl. Mech 1951, 18, 293–297. 10.1115/1.4010337.

[ref50] Liu G. R. A Step-by-Step Method of Rule-of-Mixture of Fiber- and Particle-Reinforced Composite Materials. Compos Struct 1997, 40 (3–4), 313–322. 10.1016/S0263-8223(98)00033-6.

[ref51] Thomason J. L.; Groenewoud W. M. The Influence of Fibre Length and Concentration on the Properties of Glass Fibre Reinforced Polypropylene: 2. Thermal Properties. Compos Part A Appl. Sci. Manuf 1996, 27 (7), 555–565. 10.1016/1359-835X(96)00016-4.

[ref52] Su T.; Su W.; Du C.; Huang Z.; Dong J.; Hu C. Damage Identification of Wind Turbine Blades Based on Dynamic Characteristics. Nonlinear Engineering 2022, 11 (1), 47–57. 10.1515/nleng-2022-0007.

[ref53] Chrissafis K.; Antoniadis G.; Paraskevopoulos K. M.; Vassiliou A.; Bikiaris D. N. Comparative Study of the Effect of Different Nanoparticles on the Mechanical Properties and Thermal Degradation Mechanism of in Situ Prepared Poly(ε-Caprolactone) Nanocomposites. Compos. Sci. Technol. 2007, 67 (10), 2165–2174. 10.1016/j.compscitech.2006.10.027.

[ref54] Matykiewicz D.; Barczewski M.; Michałowski S. Basalt Powder as an Eco-Friendly Filler for Epoxy Composites: Thermal and Thermo-Mechanical Properties Assessment. Compos B Eng. 2019, 164, 272–279. 10.1016/j.compositesb.2018.11.073.

[ref55] Gottlieb E.; Matyjaszewski K.; Kowalewski T. Polymer-Based Synthetic Routes to Carbon-Based Metal-Free Catalysts. Adv. Mater. 2019, 31 (13), 1804626 10.1002/adma.201804626. 30368931

[ref56] Li X.; Ji X.; Qin A.; He C. The Plasticized Spinning and Cyclization Behaviors of Functionalized Carbon Nanotube/Polyacrylonitrile Fibers. RSC Adv. 2015, 5 (64), 52226–52234. 10.1039/C5RA05696A.

[ref57] Sun S.; Zhou F.; Pan F.; Wang M.; Mao J. Hydrazine Hydrate-Initiated GO/PAN Composite Fiber Cyclization Reaction to Achieve Chemical Stabilization of PAN. ACS Sustain Chem. Eng. 2023, 11, 14481–14486. 10.1021/acssuschemeng.3c03476.

