# Supplementary material for: Coaxial Layered Fiber Spinning for Wind Turbine Blade Recycling
Source: ACS Sustain Chem Eng. 2024 Feb 13;12(8):3243–55. doi: 10.1021/acssuschemeng.3c07484 (PMC10900510; doi:10.1021/acssuschemeng.3c07484)
Supplement: Supplementary file 1 — sc3c07484_si_001.pdf [file sc3c07484_si_001.pdf]

Coaxial Layered Fiber Spinning for Wind Turbine Blade Recycling

Varunkumar Thippanna[a], Arunachalam Ramanathan[a], Dharneedar Ravichandran[a], Abhinav Chavali[b], Barath Sundaravadivelan[c], Abhishek Saji Kumar[b], Dhanush Patil[a], Yuxiang Zhu[a], Rajesh Buch[d], Maryam Al-Ejji[e], Mohammad K. Hassan[e], Lindsay R. Bick[f], Martin Taylor Sobczak[f], Kenan Song[g]

[a] Manufacturing Engineering, School of Manufacturing Systems and Networks (MSN), Ira A. Fulton Schools of Engineering, Arizona State University (ASU), Mesa, AZ, USA 85212
[b] Materials Science and Engineering, School for Engineering of Matter, Transport and Energy (SEMTE), Ira A. Fulton Schools of Engineering, Arizona State University (ASU), Tempe, AZ, USA 85287
[c] Mechanical Engineering, The School of Engineering of Matter, Transport and Energy (SEMTE), Ira A. Fulton Schools of Engineering, Arizona State University (ASU), Tempe, AZ, USA 85287
[d] Business Development Director, Rob and Melani Walton Sustainability Solutions Service, Arizona State University (ASU), Tempe, AZ, USA, 85287
[e] Center for Advanced Materials, Qatar University, P.O. BOX 2713, Doha, Qatar
[f] Mechanical Engineering, College of Engineering, University of Georgia (UGA), 302 E. Campus Rd., Athens, 30602
[g] Associate Professor of Mechanical Engineering, College of Engineering, University of Georgia (UGA), 302 E. Campus Rd., Athens, 30602 & adjunct professor at the School of Manufacturing Systems and Networks (MSN), Ira A. Fulton Schools of Engineering, Arizona State University (ASU), Mesa, AZ, USA 85212

Corresponding author, Email: kenan.song@uga.edu

Number of Pages: 19

Number of Figures: 9

Number of Tables: 7

Table of Contents

1. Mechanical recycling of Wind Turbine blades. 5

2. Fiber drawability as a function of drawing conditions 6

3. Fiber mechanical properties 9

3.1 Weibull analysis for defect density comparison within fibers 10

3.2 Numerical simulation and computational modeling for theoretical prediction of mechanical properties 12

4. Fiber thermal properties 14

5. Fiber morphologies 17

6. Heat treatment (stabilization) 18

7. References 19

Table of Figures

Figure S1. (a) Optical image of final recycled waste materials containing mixture of glass fibers, resins and other core materials, (b) TGA for the glass fiber reinforced plastics to determine the glass fibers concentration, (c) Glass fiber particle size (length) distribution with an average count of 35 fibers, (d) shows the diameter of glass fibers and (e) aspect ratio of the glass fibers. 6

Figure S2. Mechanical properties of heat-drawing fibers 10

Figure S3. Weibull analysis of the fitted strength for (a1) PAN, and (a2) PAN-0.1wt% GF and of the fitted modulus for (b1) PAN, and (b2) PAN-0.1wt% GF. The fitted values are summarized in **Table S4** 12

Figure S4. Computational depiction of stress variation in the 3 layered composite using FEA through ABAQUS software for 0.1wt% GF with varying modulus of 40GPa – 60GPa respectively in (a_1), (a_2) &(a_3) and for 1wt% GF with varying modulus of 40GPa – 60GPa respectively as seen in (b_1), (b_2) & (b_3). The displacement contours for the fibers at a constant strain rate of 50 μm/min for 0.1wt% GF (c_1) and 1wt% GF (c_2). Tuning the bulk PAN's modulus between 14.5 and 17.5 GPa with a GF modulus of 60 GPa (d) would generate the composite modulus and the fitted equation will generate an accurate PAN modulus matching the composite stiffness. 14

Figure S5. DSC curves of different draw ratio fiber types (a_1), (a_2) & (a_3) in nitrogen, followed by their re-runs on the air (b_1), (b_2) & (b_3). 16

Figure S6. Morphologies of 10% PAN/10%PAN-200 wt% GF fiber fractured surfaces 18

Figure S7. Morphologies of 12% PAN/12%PAN-200 wt% GF fiber fractured surfaces 18

Figure S8. Morphologies of 12% PAN/12%PAN-10 wt% GF fiber fractured surfaces 18

Figure S9. Heat treatment (Stabilization process) in the presence of air for the highest draw ratio fibers. 19

Table of Tables:

Table S1. Fiber type of increasing draw ratios and their thickness 7

Table S2. Summarized literature studies on the nanoparticles and their effect on polymer crystallization behavior 8

Table S3. Summary of fiber drawing and mechanical properties of heat-drawing fibers 10

Table S4. Weibull modulus and strength fitted values 12

Table S5. Peak temperature for the different fiber types in air and N_2 atmosphere during the stabilization process 15

Table S6. Peak temperature of PAN-10wt% GF composite fiber at different heating rates. 16

Table S7. Activation energies of PAN and their composites determined from Kissinger method from literature reports as compared to our research. 17

1. Mechanical recycling of Wind Turbine blades.

Wind turbine blades (WTBs) are composed of a polymer matrix—crafted from thermoplastics or thermosetting plastics—reinforced by fibers like glass, carbon, or aramid. They integrate a lightweight core such as balsa wood or foam and polymer coatings, shielding against environmental factors and enhancing strength-to-weight ratios. Predominantly, WTBs are constructed using glass fiber-reinforced polymer composites (GFRPs), where fibers and polymers combine to form the primary composition.[1] These GFRP were mechanically recycled before the addition into the PAN/DMF solution through shredding, crushing, milling, and sieving (via the mesh 40). Image J was used to predict the average particle size (length) of the process GFRP and was found to be 38 μm (**Figure S1**). Obtaining pure glass fibers from mechanical recycling processes is challenging due to the inherent difficulty in separating resin from the fibers completely.[2]

Mechanical recycling of wind turbine blades offers notable advantages. It consumes less energy compared to chemical methods, resulting in a reduced carbon footprint. Simplicity and lower costs characterize mechanical processes like shredding and sorting. These methods better maintain material purity and original properties. They also pose fewer environmental risks compared to chemical approaches, often involving hazardous substances. Additionally, mechanical recycling boasts established technologies, easier industrial implementation, and greater flexibility in handling diverse blade materials. Overall, it stands as a more energy-efficient, cost-effective, and environmentally friendly option for handling end-of-life turbine blades.[3,4] The mechanical properties of composite fibers can indeed be notably influenced by the concentration of glass fibers in the PAN/DMF solution. When glass fibers constitute the majority of the composite material, their characteristics play a dominant role in determining mechanical performance, such as strength[5], stiffness, and durability[6].

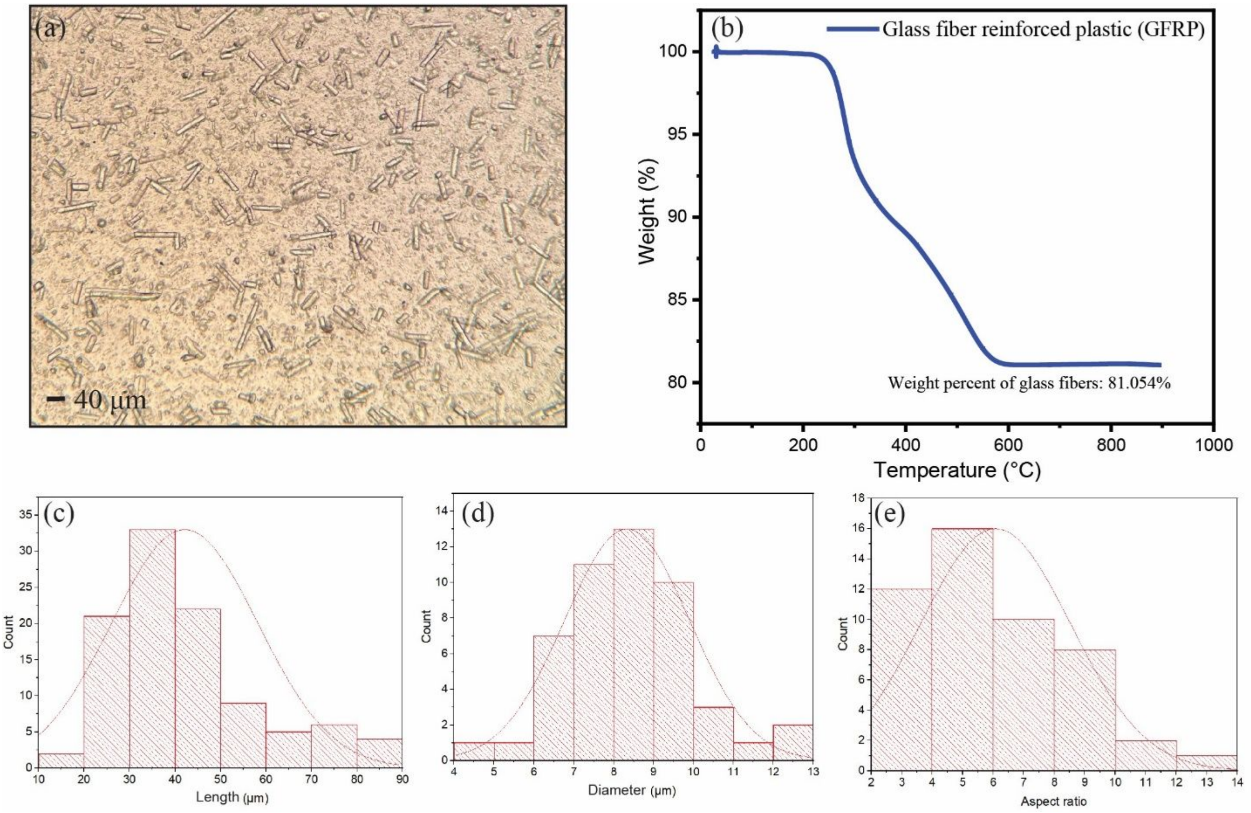

Figure S1. *(a) Optical image of final recycled waste materials containing mixture of glass fibers, resins and other core materials, (b) TGA for the glass fiber reinforced plastics to determine the glass fibers concentration, (c) Glass fiber particle size (length) distribution with an average count of 35 fibers, (d) shows the diameter of glass fibers and (e) aspect ratio of the glass fibers.*

2. Fiber drawability as a function of drawing conditions

Table S1. *Fiber type of increasing draw ratios and their thickness*

Fiber type	Spinning medium	Max speed (sec)	Min speed (sec)	Individual draw ratio	Total Draw ratio	Thickness (microns)
12wt%PAN	Water (85°C)	**3.44**	**1.74**	**2.00**	**2.00**	**378**
		3.44	1.55	2.22	4.50	320
		3.76	2.50	1.52	6.50	193
	Oil (120°C)	3.33	1.52	2.20	14.50	117
	Oil (135°C)	3.30	2.28	1.45	21.00	95
	Oil (145°C)	**4.27**	**2.77**	**1.54**	**32.50**	**83**
12wt%PAN/ 0.1wt%PAN/GF	Water (85°C)	**2.34**	**1.40**	**1.67**	**1.67**	**-**
		2.35	1.51	1.55	2.59	-
		3.50	1.20	2.92	7.55	-
	Oil (120°C)	2.13	1.20	1.77	13.40	-
	Oil (135°C)	4.15	1.81	2.30	30.82	-
	Oil (145°C)	4.05	2.58	1.57	48.38	-
		**4.92**	**3.78**	**1.30**	**63.00**	**54**
12wt%PAN/ 1.0wt%PAN/GF	Water (85°C)	3.12	1.51	2.00	2.00	**-**
		2.73	1.60	1.70	3.50	-
		2.28	1.47	1.55	5.50	-
	Oil (120°C)	3.04	1.59	2.00	10.50	-
	Oil (135°C)	3.45	1.79	2.00	20.00	-
	Oil (145°C)	**3.62**	**1.78**	**2.00**	**41.00**	**65**
12wt%PAN/ 2.5wt%PAN/GF	Water (85°C)	**2.35**	**1.29**	**2.00**	**2.00**	**-**
		3.04	1.25	2.43	4.50	-
		2.20	1.35	1.62	7.25	-
	Oil (120°C)	2.62	1.31	2.00	14.50	-
	Oil (135°C)	3.66	2.43	1.50	21.75	-
	Oil (145°C)	4.05	2.02	2.00	43.50	60
12wt%PAN/ 10wt%PAN/GF	Water (85°C)	**3.35**	**1.68**	**2.00**	**2.00**	**-**
		3.15	2.07	1.52	3.04	-
		2.45	1.69	1.45	4.40	-
	Oil (120°C)	2.34	1.30	1.80	7.92	-
	Oil (135°C)	3.31	1.91	1.73	13.72	-
	Oil (145°C)	2.70	1.70	1.59	21.8	-
		2.77	1.92	1.44	31.45	78
12wt%PAN/ 50wt%PAN/GF	Water (**85**°C)	**2.64**	**1.67**	**1.60**	**1.50**	**337**
		3.08	1.50	2.05	3.00	250
		2.26	1.56	1.45	4.50	176
	Oil (120°C)	2.25	1.50	1.50	7.00	125
	Oil (135°C)	3.05	2.11	1.45	10.50	100
	Oil (145°C)	2.70	1.90	1.42	14.50	94
		**3.13**	**2.70**	**1.16**	**17.00**	**83**
12wt%PAN/ 100wt%PAN/GF	Water (**85**°C)	**2.46**	**1.56**	**1.60**	**1.50**	**453**
		1.99	1.44	1.38	2.25	373
		2.08	1.42	1.50	3.25	344
	Oil (120°C)	2.23	1.60	1.40	4.50	247
	Oil (135°C)	2.73	1.34	2.04	9.00	211
	Oil (145°C)	2.56	1.65	1.55	14.50	192
		**2.40**	**1.22**	**1.98**	**28.00**	**144**
12wt%PAN/ 200wt%PAN/GF	Water (**85**°C)	**2.03**	**1.15**	**1.75**	**2.00**	**498**
		2.01	1.03	2.00	3.50	283
		1.93	1.10	1.75	6.00	243
	Oil (120°C)	2.93	1.69	1.75	10.50	235
	Oil (135°C)	4.28	2.30	1.80	20.00	227
	Oil (145°C)	**3.78**	**2.62**	**1.50**	**28.00**	**224**
10wt%PAN/ 200wt% PAN/GF	Water (**85**°C)	**2.25**	**1.06**	**2.12**	**2.00**	**344**
		2.02	1.21	1.67	3.50	300
		2.05	1.26	1.63	5.5	298
	Oil (120°C)	3.35	1.81	1.85	10.5	178
	Oil (135°C)	2.47	1.76	1.40	15.00	164
	Oil (145°C)	**2.88**	**2.15**	**1.34**	**20.00**	**156**

The Polymer crystallization behavior of the polymer composites and the alignment of the glass fibers. The summary of the references is mentioned in the **Table S2**

Table S2. *Summarized literature studies on the nanoparticles and their effect on polymer crystallization behavior*

Year	Title	Focus on polymer crystallization	Reference
2011	Nucleation and Growth of Zinc Sulfide Nanoparticles in Ultrathin Polymer Films by Layer-by-Layer Polyionic Assemblies.	The article explores how the polymer crystallization behavior is affected by the nucleation and growth of these nanoparticles within the polymer matrix. It investigates the alignment, distribution, and interaction of the nanoparticles within the polymer films, elucidating their impact on crystallization kinetics and morphological aspects of the polymer-nanoparticle composite. The research also shed light on how these nanoparticles influence the polymer's structural characteristics and crystalline properties.	7
2005	Surface Assisted Nucleation and Growth of Polymer Latexes on Organically Modified Inorganic Particles	The article delves into the influence of surface-modified inorganic particles on polymer crystallization behavior and nanoparticle alignment within the polymer matrix. Investigating these modifications' role as nucleation sites for polymer growth, it details their impact on crystallization kinetics, alignment, and nanocomposite morphology. The research likely offers insights into manipulating polymer crystallization and nanoparticle alignment via tailored surface modifications, ultimately influencing material properties and performance. This exploration likely provides strategies to control or enhance polymer behavior through precisely engineered alterations to inorganic particle surfaces, shaping the resulting nanocomposite's characteristics	8
2013	Structure and morphology control in crystalline polymer–carbon nanotube nanocomposites	This study delves into the intricate relationship between carbon nanotubes (CNTs) and polymer crystallization. It uncovers CNTs' role as catalysts for polymer crystallization while hindering crystal growth. Variations in CNT characteristics and sample preparation make uniform quantification across research groups challenging. The article spotlights nanohybrid shish-kebab structures, illustrating CNTs' influence on nucleation density, temperature gradients, and periodic polymer crystallization along CNT axes. Comparisons with carbon fiber-polymer systems highlight trans-crystallization phenomena, emphasizing CNTs' distinct impact on polymer chain orientation and crystallization behavior. Ultimately, this study offers new perspectives on enhancing material properties through controlled polymer crystallization mediated by CNTs.	9
2006	Polymer crystallization-driven, periodic patterning on carbon nanotubes	The article explores polymer solution crystallization for controlled carbon nanotubes (CNT) surface modification, resulting in nano-hybrid shish-kebab (NHSK) structures with aligned PE single-crystal lamellae around single walled nanotubes (SWNTs). This NHSK formation occurs without	10

		shear flow, revealing SWNTs as effective nucleation surfaces. The study unveils a new route for periodic CNT functionalization (PCCF), vital for tailored electrical and optical applications. PCCF enables tunable periodic functionalization, maintaining CNT integrity, while NHSK formation depends on crystallization conditions and CNT structures, impacting CNT dispersion and separation efficiency. This approach holds promise for precise CNT alignment and controlled crystallization behavior within polymer matrices.	
2023	Fundamentals of Crystalline Evolution and Properties of Carbon Nanotube-Reinforced Polyether Ether Ketone Nanocomposites in Fused Filament Fabrication	The research examines non-isothermal cold crystallization of polyetheretherketone (PEEK)was studied in the presence of carbon nanotubes (CNTs), PEEK-CNT nanocomposites after reaching the glass-transition temperature (162-170°C). This less-explored phenomenon, linked to rapid cooling in additive manufacturing, swiftly immobilizes the polymer, curtailing crystallization. CNT inclusion in PEEK reduces cold crystallization due to nucleating effects and limited polymer mobility. Controlled cooling halts this phenomenon. Additionally, CNTs elevate crystallization temperatures, prompting more crystallites and rapid crystallization, advantageous in FFF printing and injection molding. Despite enhanced nucleation, controlled cooling and CNT spatial confinement reduce crystallinity, notably in higher CNT concentrations. CNT-induced nucleation and confinement dynamics affect crystallization: lower concentrations promote nucleation, while higher concentrations restrict polymer movement, decreasing crystallinity. This spatial confinement intensifies with rising nanoparticle concentrations. Understanding this interplay between CNTs, cooling rates, and crystallization provides crucial insights for optimizing manufacturing methods involving polymer-CNT nanocomposites.	11

3. Fiber mechanical properties

It is crucial to carefully control the PAN concentration during the spinning process to minimize the formation of voids and defects and ensure the production of fibers with optimal mechanical properties. The decrease in the concentration of PAN in the spinning solution not only affects the number of polymer chains available for fiber formation but can also impact the formation of voids and defects in the fibers. The presence of voids and defects can weaken the mechanical properties of the fibers and reduce their overall strength as we see from (**Table S3)** for 12wt%PAN and 10wt%PAN with the same 200wt %GF. These voids and defects can act as stress concentrators, leading to localized failure under mechanical loading.

Table S3. *Summary of fiber drawing and mechanical properties of heat-drawing fibers*

Fiber type	Spinning medium	Total Draw ratio	Diameter (microns)	Heat-drawing fibers		
				**Youngs Modulus (GPa)**	**Tensile Strength (MPa)**	**Elongation at break (%)**
12wt%PAN	Water (85°C)	**2.00**	**378**	1.05 ± 0.45	19.60 ± 3.10	2.23 ± 0.34
		4.50	320	1.21 ± 0.45	41.50 ± 5.80	9.55 ± 2.55
		6.50	193	2.93 ± 0.85	127.66 ± 4.32	17.37 ± 0.65
	Oil (120°C)	14.50	117	5.16 ± 1.52	22348 ± 21.5	11.26 ± 2.83
	Oil (135°C)	21.00	95	6.44 ± 0.42	235.84 ± 11.28	9.82 ± 0.61
	Oil (145°C)	**32.50**	**83**	14.55 ± 1.82	460.00 ± 15.00	7.40 ± 1.57
12wt% PAN/ 0.1wt% GF	Water (85°C)	1.50	-	1.10 ± 0.24	28.10 ± 9.49	13.89 ± 0.034
		2.50	-	1.50 ± 0.12	44.70 ± 11.11	3.69 ± 0.49
		7.50	-	3.65± 0.84	150.21 ± 11.50	18.91 ± 1.57
	Oil (120°C)	13.50	-	3.48 ± 0.65	160.53 ± 4.54	10.83 ± 2.69
	Oil (135°C)	31.00	-	9.23 ± 1.14	327.40 ± 28.65	6.69 ± 0.66
	Oil (145°C)	48.50	-	11.88 ± 1.01	418.21 ± 39.50	6.98 ± 1.05
		63.00	54	17.12 ± 3.42	572.25 ± 31.00	5.72 ± 0.06
10wt%PAN/ 200wt%PAN/GF	Water (**85**°C)	**2.00**	**344**	0.97 ± 0.16	9.20 ± 0.58	1.02 ± 0.27
		3.50	300	1.44 ± 0.22	21.18 ± 5.70	1.83 ± 0.98
		5.50	298	1.83 ± 1.0	41.46 ± 3.95	8.14 ± 1.76
	Oil (120°C)	10.50	178	1.67 ± 0.40	58.37 ± 6.70	8.55 ± 1.51
	Oil (135°C)	15.00	164	2.86 ± 0.55	85.03 ± 0.75	6.80 ± 0.98
	Oil (145°C)	**20.00**	**156**	3.42 ± 0.18	108.10 ± 4.50	7.76 ± 0.26

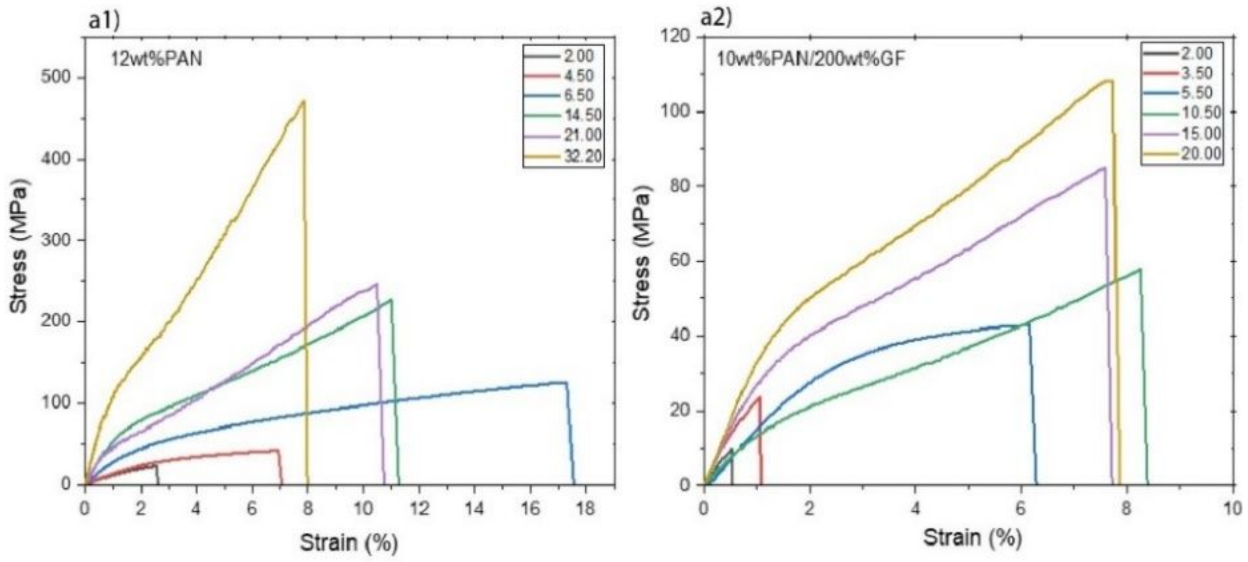

Figure S2. *Mechanical properties of heat-drawing fibers*

3.1 Weibull analysis for defect density comparison within fibers

Statistical Distribution for Tensile Strength and Young's Modulus:

The Weibull distribution is a pivotal tool used in materials science to evaluate the strengths of brittle materials. It can help identify the distribution of strengths and predict the probability of failure under different stress conditions, facilitates precise comparisons between various materials, and holds substantial significance in appraising material strength and engineering component design. Its application is fundamental in ensuring the reliability and integrity of engineered systems.[12] The model predicts that in experiments, the defects' severity would follow a Poisson distribution. The most serious defects in a fiber are distributed and propagated to the weakest point in the fiber, which determines the fiber's strength. **Equations S1 and S2** provide the two-parameter Weibull distribution's cumulative distribution function, P(σ).

$$P(\sigma) = 1 - exp\left[-\left(\frac{\sigma}{x_0}\right)\beta\right] \quad \textbf{\textit{(Equation S1)}}$$

$$ln\left(ln\frac{1}{1-P(\sigma)}\right) = \beta[ln(\sigma) - ln(x_0)] \quad \textbf{\textit{(Equation S2)}}$$

P(σ) is the probability of failure, σ is the failure strength of the fiber, x_0 is the scale parameter (unit *Pa*), and β is the scale parameter (no dimension), with different variations in strength distribution.[13] The above equations are used widely for a single set of fibers to fit the experimental data. A linear plot is obtained from **Equation 2**, which will give the shape parameter (β) and scale parameter (x_0), based on the fiber failure strength (σ).

In equation 3, the lower mean squared error is used to estimate the probability of failure, where *i* is the failure rank (σ_1 is the lowest rank failure stress, σ_N is the highest rank failure stress) and N is the total number of measurements. The Weibull distribution's formulas bear significant physical relevance. A higher β indicates enhanced fiber stability. According to **Equation 1**, with a large β, $\sigma<x_0$ yields *P=0* (no failure), and $\sigma>x_0$ yields P=1 (failure), indicating less or fewer defects. Conversely, a smaller β results in a probabilistic failure distribution, signifying axis inhomogeneity within fiber structures. This insight is crucial for assessing materials' integrity.

$$P(\sigma) = \frac{i - 0.5}{N} \quad \textbf{\textit{(Equation S3)}}$$

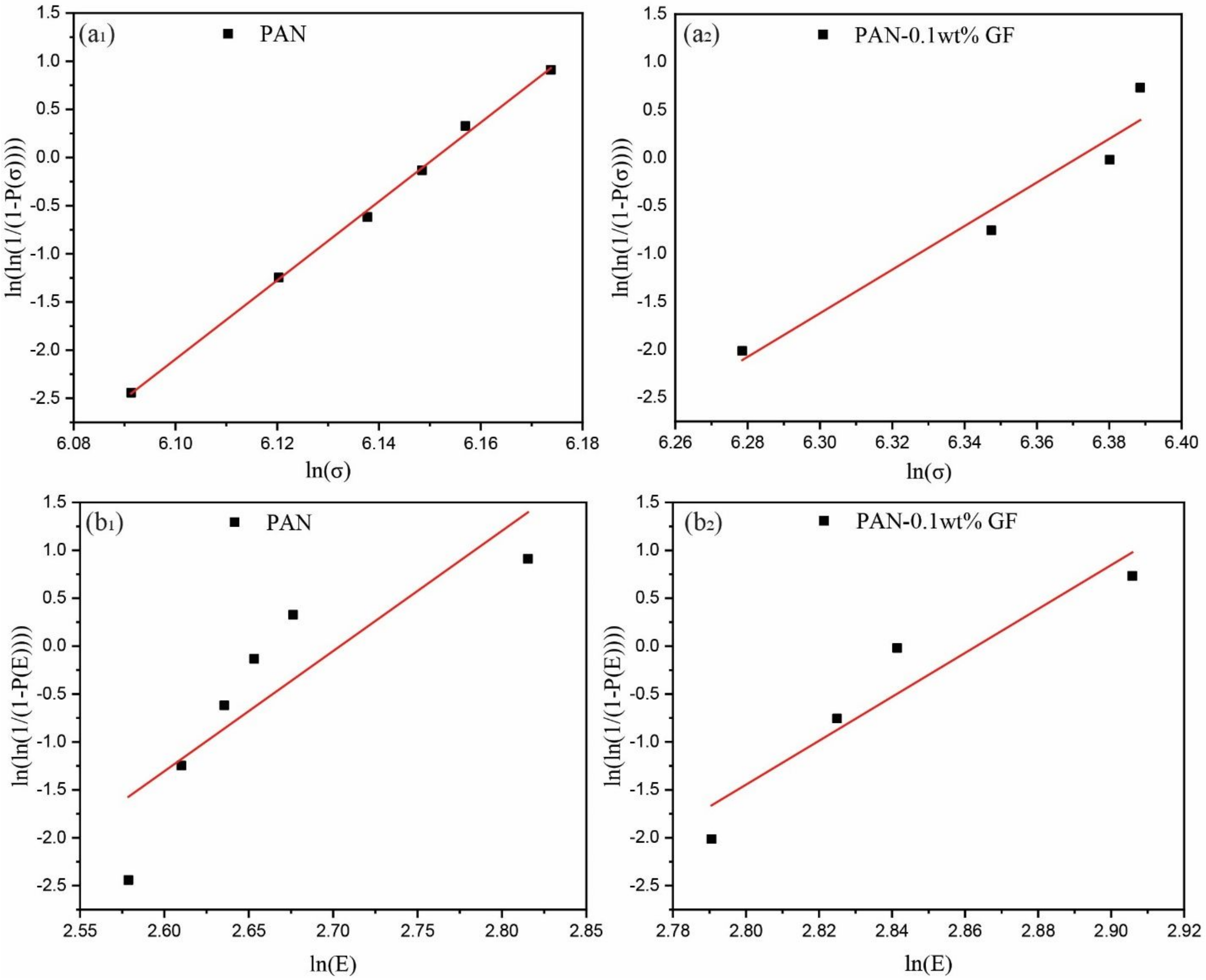

Figure S3. *Weibull analysis of the fitted strength for (a1) PAN, and (a2) PAN-0.1wt% GF and of the fitted modulus for (b1) PAN, and (b2) PAN-0.1wt% GF. The fitted values are summarized in* ***Table S4***

Table S4. *Weibull modulus and strength fitted value*s

**Fiber name**	**Weibull Analysis**			
	**Modulus fitting**		**Strength fitting**	
	x_0 (GPa)	**β**	x_0 (MPa)	**β**
PAN	14.94	**12.54**	469.21	40.98
PAN – 0.1wt% GF	17.52	**22.91**	584.87	22.75

3.2 Numerical simulation and computational modeling for theoretical prediction of mechanical properties

This study employed Abaqus/Explicit, a versatile finite element modeling software renowned for its robust material modeling capabilities and comprehensive mechanical component analysis (Abaqus 6.11 SIMULIA). The simulation emulated the Uniaxial tensile test configuration conducted on PAN/GF composite. Varied parameters included fiber volume fraction, pure PAN elastic modulus, and GF modulus, ranging from 14.5 GPa to 17.5 GPa and 40 GPa to 60 GPa, respectively. This aimed to ascertain the overall longitudinal modulus of the composite. For PAN elastic modulus determination in 0.1wt% and 1wt% GF composites post-reinforcement and composite integration, a range of modulus values was obtained. These were then interpolated with experimental values of 17.12 GPa and 15.87 GPa

In this study, the composite's behavior was analyzed under uniform strains at the macroscopic level, denoted as macrostrains. Correspondingly, the stresses at this scale are referred to as macro stresses. At the micro level, within the Representative Volume Element (RVE), actual stresses may exhibit spatial variation. Macro stresses represent the average stress needed to induce a specific state of macro-deformation, and their determination is achievable through finite element analysis.

A micromechanical elastic analysis of a unidirectional PAN/GF composite was conducted using the finite element method. Several assumptions were integrated into the geometric and parameter modeling process:

- Uniformity of elastic moduli and tensile strength within the fibers along the RVE.
- Mechanical properties vary solely in the axial direction of force application.
- Homogeneity in the composite layer where glass fibers were experimentally reinforced.
- Assumption of a void-free composite, disregarding the presence of particle-level polymer inclusions.
- The geometry depicted in the contours represents a scaled-up version of the experimental specimen. This measure was implemented to ensure mesh linearity and mitigate potential distortions.

A uniaxial tensile test configuration was replicated, applying strain load exclusively in the z-direction while constraining the rare and front ends in all other directions, resulting in zero shear strain. Consequently, only longitudinal stresses and strains were observed, simplifying the scenario into a uniaxial problem. This simplification assumed linear elastic behavior and overlooked Poisson's effect, implying no lateral or radial geometry changes upon load application. A tetrahedral element mesh, comprising 90,000 elements, was employed. The mesh was meticulously generated, ensuring a fine and linear structure at the cylinder's load application end. PAN moduli were evaluated and simulated based on the provided parameters. The stress distribution within the fiber geometry for a 3-layered composite with a varying modulus of 40GPa – 60GPa for 0.1wt% GF **(Figures S4a_1-a_3)** and 1wt% GF **(Figures S4b_1-b_3)** which was graphically represented as displacement was applied to the nearside, while the offside remained fixed. Notably, an inverse relationship between the GF modulus and the innate modulus of PAN fibers was observed. This deviation in PAN elastic modulus, post its incorporation into the composite compared to the pure PAN sample, arose from bonding interactions and agglomeration formation after GF reinforcement. These interactions created local stress concentrations, subsequently reducing the overall composite's elasticity, and altering the load transfer dynamics between the fiber and matrix. Displacement contours, under the specified conditions and parameters with a strain rate of 50 μm/min, were depicted (**Figures S4c_1-c_2**). These visualizations illustrated that as one moved away from the nearside towards the offside, the impact of the displacement load markedly diminished, given the complete fixation of the offside end. This information is instrumental in validating experimental tests, particularly in the elastic portion of the strain distribution along the composite's geometry. Furthermore, the FEA analysis comprehensively accounted for the localized distribution of stress and strain across the geometry's cross-section throughout the uniaxial tensile test, up until reaching the test's elastic limit. Our determination of the intrinsic modulus of PAN shown in **Figure S4d** was achieved by fitting the composite modulus to the experimental results **(Table 2).**

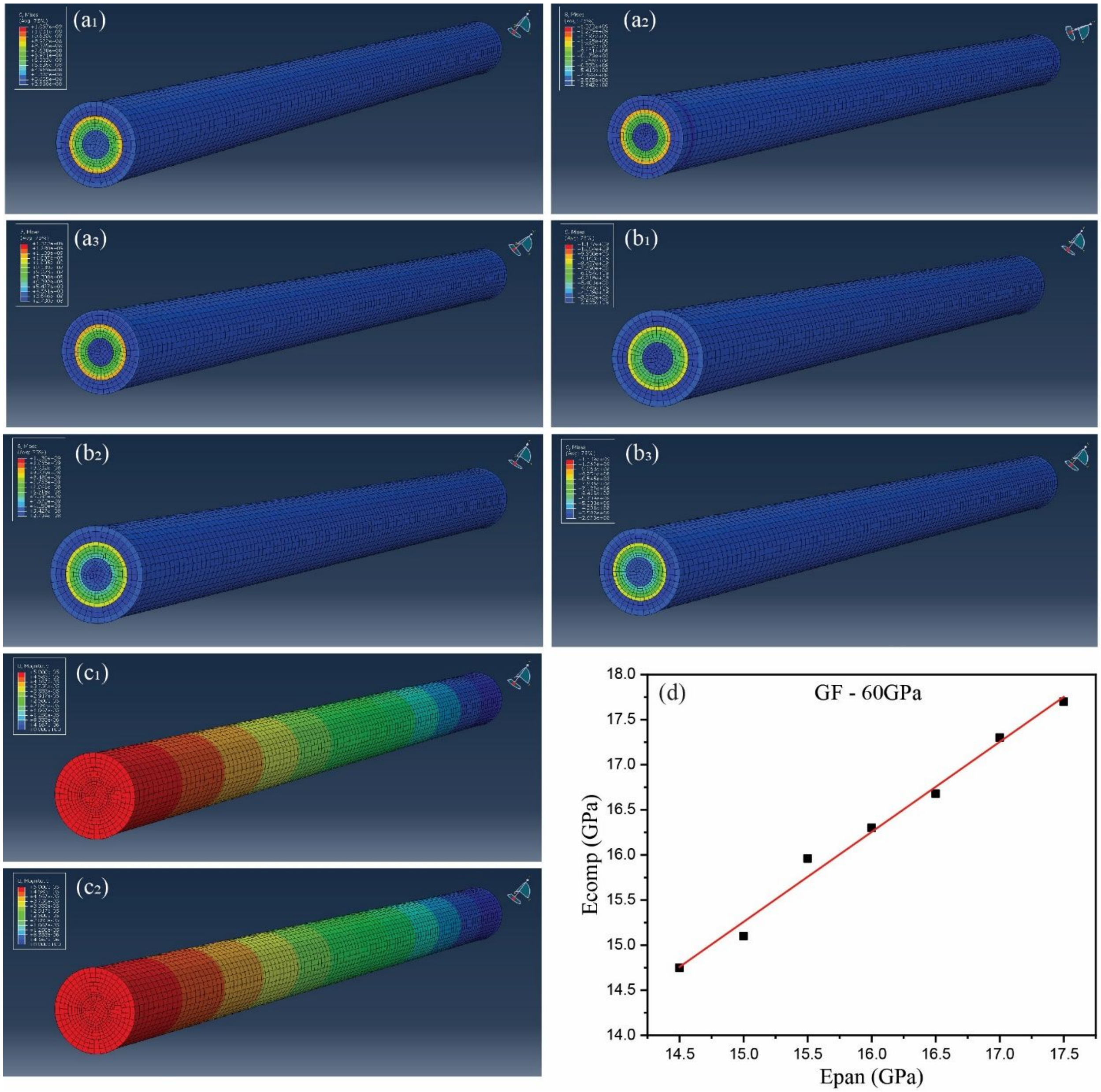

Figure S4. *Computational depiction of stress variation in the 3 layered composite using FEA through ABAQUS software for 0.1wt% GF with varying modulus of 40GPa – 60GPa respectively in (a_1), (a_2) &(a_3) and for 1wt% GF with varying modulus of 40GPa – 60GPa respectively as seen in (b_1), (b_2) & (b_3). The displacement contours for the fibers at a constant strain rate of 50 µm/min for 0.1wt% GF (c_1) and 1wt% GF (c_2). Tuning the bulk PAN's modulus between 14.5 and 17.5 GPa with a GF modulus of 60 GPa (d) would generate the composite modulus and the fitted equation will generate an accurate PAN modulus matching the composite stiffness.*

4. Fiber thermal properties

Table S5. *Peak temperature for the different fiber types in air and N_2 atmosphere during the stabilization process*

Fiber type	Total draw ratio (DR)	Air	Nitrogen
		T_{oxi} (°C)	T_{cyc} (°C)
12wt%PAN	**2.00**	270.80	272.12
	4.50	275.60	273.95
	6.50	279.18	280.21
	14.50	284.61	287.28
	21.00	287.10	286.97
	**32.50**	287.76	290.47
12wt%PAN/ 50wt%PAN/GF	**1.50**	272.46	268.02
	3.00	274.28	273.09
	4.50	277.87	279.39
	7.00	282.34	284.50
	10.50	284.12	285.91
	14.50	284.60	285.50
	**17.00**	288.23	291.37
12wt%PAN/ 100wt%PAN/GF	**1.50**	266.92	267.11
	2.25	271.63	271.92
	3.25	274.29	273.32
	4.50	281.97	280.00
	9.00	282.71	281.34
	14.50	284.79	286.48
	**28.00**	288.52	284.31
12wt%PAN/ 200wt%PAN/GF	**2.00**	283.79	289.05
	3.50	284.23	289.74
	6.00	284.91	290.04
	10.50	288.05	292.52
	20.00	293.34	296.70
	**28.00**	291.83	298.02
10wt%PAN/ 200wt%PAN/GF	**2.00**	275.94	286.81
	3.50	274.78	283.35
	5.5	278.53	286.10
	10.5	287.98	289.38
	15.00	286.18	288.24
	**20.00**	286.08	285.04

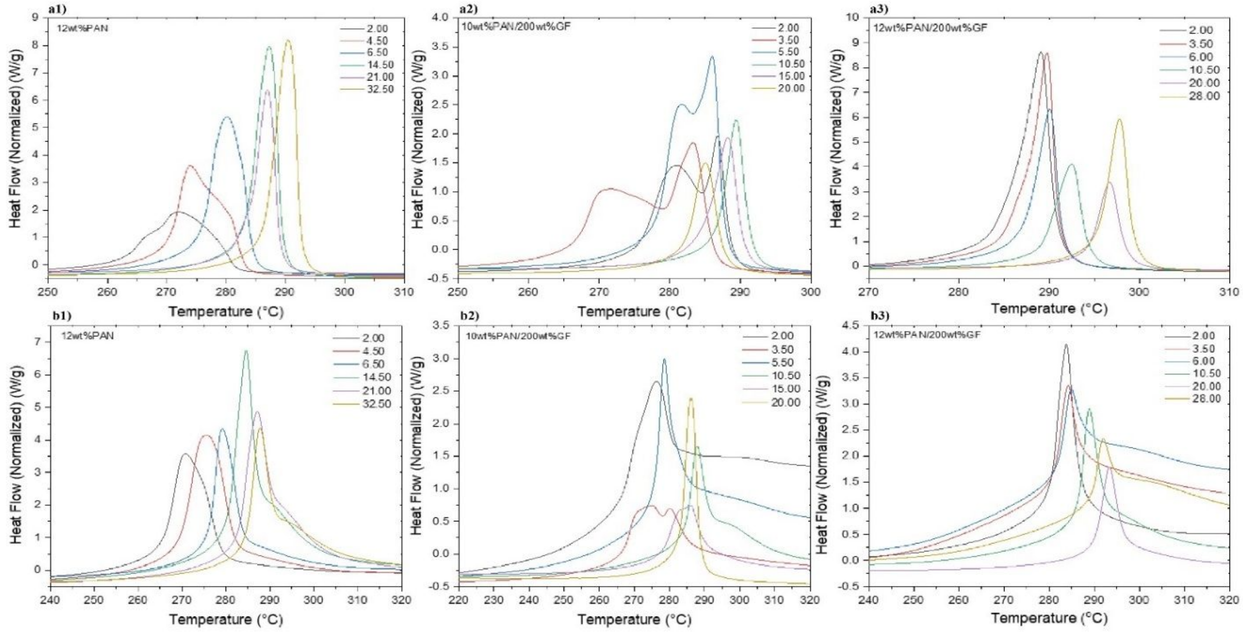

Figure S5. *DSC curves of different draw ratio fiber types (a_1), (a_2) & (a_3) in nitrogen, followed by their re-runs on the air (b_1), (b_2) & (b_3).*

Table S6. *Peak temperature of PAN-10wt% GF composite fiber at different heating rates.*

Fiber type	Heating rates (°C/min)	Peak temperature (N_2 -°C)	Enthalpy (J/g)
**PAN – 10wt% GF**	5	293.90	282.96
	10	305.54	189.24
	15	315.12	181.18
	20	321.16	176.60
	25	326.09	147.97

Activation energy (Ea) is the energy barrier that prevents polymer migration from the melt to the crystal surface. A higher activation energy suggests a higher energy barrier for the polymer to cross over from the liquid state to the crystal surface, it denotes greater difficulty in melt crystallization.[14] The E_a of cyclization mentioned in **Table S7**.

In some literature reports, the introduction of CNTs was observed to decrease the activation energy of the cyclization reaction. This suggests that CNTs might facilitate and promote the cyclization reaction, potentially occurring near the vicinity of the CNTs. In the presence of CNTs, the nitrile groups within PAN chains can undergo intramolecular reactions, forming rings within a single molecule or bonds between different macromolecules. These chemical reactions contribute to chemically induced shrinkage in the material.[15] In another report, the activation energies for oxidation and crosslinking PAN/GNP-based composite fibers showed much lower values than the 15%PAN fibers. Since oxidation and crosslinking require an oxidative environment to proceed, one explanation was the faster oxygen diffusion in the composite fibers, indicating a void-containing structure.[16] Similarly, the addition of 40 wt% CNCs (Cellulose Nanocrystals) in PAN reduces cyclization and crosslinking activation energies by 17.5% and 19%, respectively (**Table S7**), yet oxidation activation energies remain comparable between PAN and PAN/CNC-40 fibers. Upon heating PAN/CNC-40 fibers in the air following a nitrogen atmosphere, two peaks indicative of oxidation and cross-linking reactions emerge. The presence of CNCs introduces potential additional reactions, such as CNC dehydration, depolymerization, interactions with PAN, and CNC-catalyzed reactions, enhancing the complexity of the stabilization process compared to PAN copolymer stabilization in air.[17] Also, the activation energy of oxidation and crosslinking reactions are lowered when BNNT (boron nitride nanotubes) is added to PAN fiber. The cyclization activation energy values of the two PAN/BNNT composite fibers are comparable. The presence of BNNT affects the carbon structure compared to the PAN-based fiber.[18]

***Table S7**. Activation energies of PAN and their composites determined from Kissinger method from literature reports as compared to our research.*

Polymers and their composites	Activation energies E_a (KJ/mol)			Reference
	Cyclization	Oxidation	Cross-linking	
PAN	187.5	65.7	-	[13]
PAN/CNT-3	164.3	81.3	-	
15%PAN	146.5	84.2	138.5	[14]
0%PAN/10%GNP-3	151.8	77.5	112.1	
PAN	198.5	83.5	157.7	[15]
PAN/CNC - 40	163.8	83.1	127.8	
PAN	164.2	72.8	194.0	[16]
PAN-BNNT	166.0	67.5	120.9	
PAN	144.2	157.02	-	Current research
PAN-0.1wt% GF	124.7	120.7	-	

5. Fiber morphologies

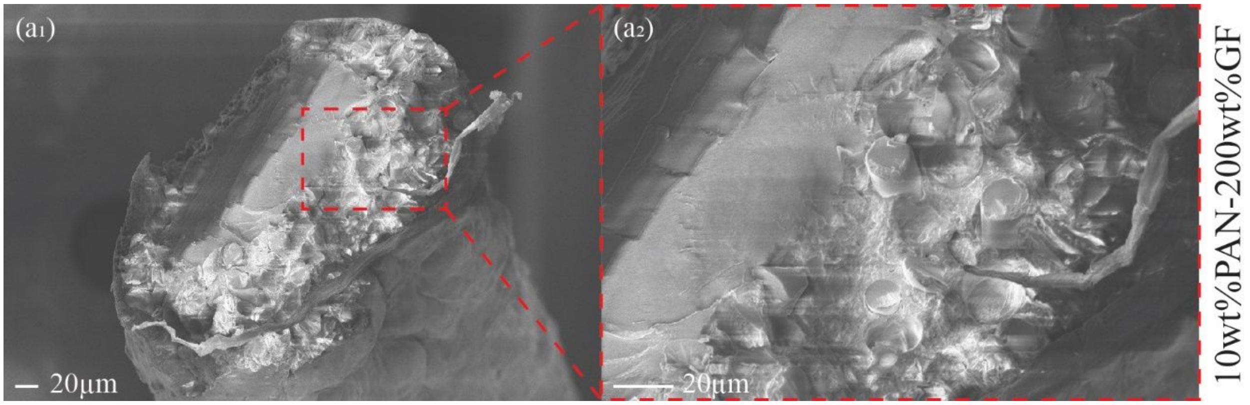

Figure S6. *Morphologies of 10% PAN/10%PAN-200 wt% GF fiber fractured surfaces*

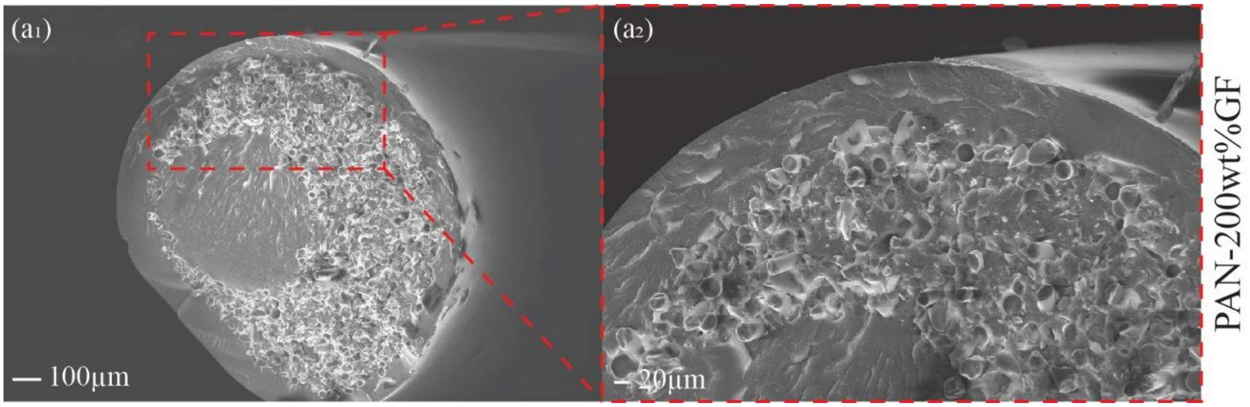

Figure S7. *Morphologies of 12% PAN/12%PAN-200 wt% GF fiber fractured surfaces*

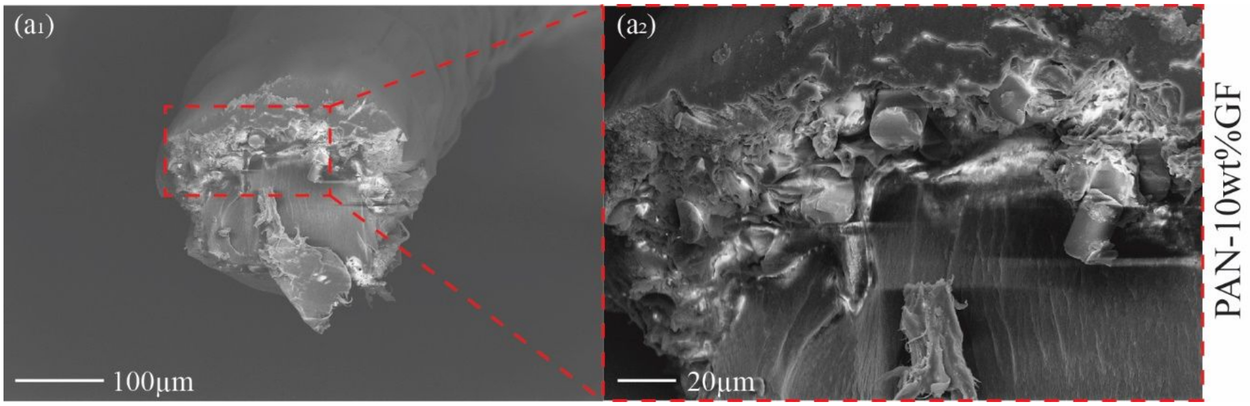

Figure S8. *Morphologies of 12% PAN/12%PAN-10 wt% GF fiber fractured surfaces*

6. Heat treatment (stabilization)

Our future studies will focus on the heat treatment of the obtained PAN and PAN composite fibers, such as the stabilization and carbonization procedures. During the stabilization process of PAN-based fibers, fiber shrinkage is a common phenomenon that is often observed. This shrinkage occurs due to the structural and chemical transformations taking place within the fiber during stabilization. The shrinkage observed during stabilization can be attributed to the release of volatile components, such as water, carbon dioxide, and other gases, as well as the rearrangement of polymer chains and the development of a more ordered and compact structure. It is important to control the shrinkage during the stabilization process to avoid distortion or damage to the fibers. Various parameters, such as temperature, heating rate, and atmosphere, can be adjusted to minimize the shrinkage and ensure the dimensional stability of the fibers.

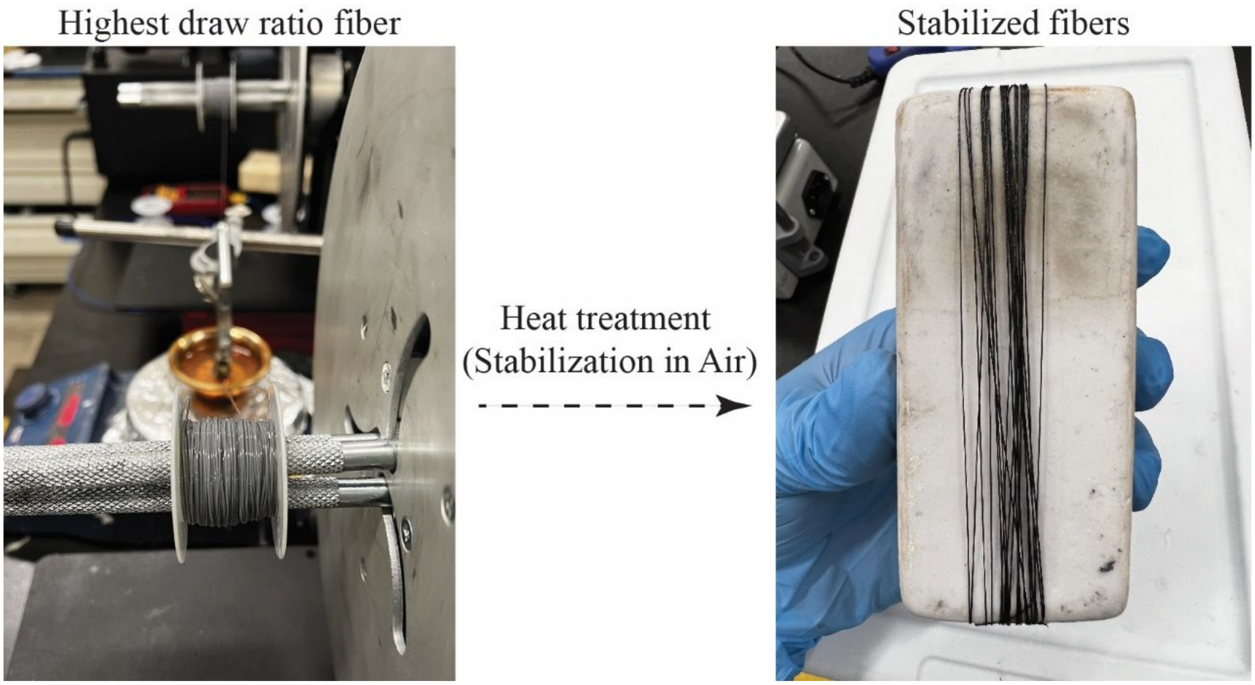

Figure S9. *Heat treatment (Stabilization process) in the presence of air for the highest draw ratio fibers.*

7. References

(1) Mumtaz, H.; Sobek, S.; Sajdak, M.; Muzyka, R.; Drewniak, S.; Werle, S. Oxidative Liquefaction as an Alternative Method of Recycling and the Pyrolysis Kinetics of Wind Turbine Blades. *Energy* **2023**, *278*, 127950.

(2) Jensen, J. P. Evaluating the Environmental Impacts of Recycling Wind Turbines. *Wind Energy* **2019**, *22* (2), 316–326.

(3) Yafei Shen; Emmanuel Apraku, S.; Yupeng Zhu. Recycling and Recovery of Fiber-Reinforced Polymer Composites for End-of-Life Wind Turbine Blade Management. *Green Chemistry* **2023**, *25* (23), 9644–9658.

(4) Giorgini, L.; Benelli, T.; Brancolini, G.; Mazzocchetti, L. Recycling of Carbon Fiber Reinforced Composite Waste to Close Their Life Cycle in a Cradle-to-Cradle Approach. *Curr Opin Green Sustain Chem* **2020**, *26*, 100368.

(5) Withers, G. J.; Yu, Y.; Khabashesku, V. N.; Cercone, L.; Hadjiev, V. G.; Souza, J. M.; Davis, D. C. Improved Mechanical Properties of an Epoxy Glass–Fiber Composite Reinforced with Surface Organomodified Nanoclays. *Compos B Eng* **2015**, *72*, 175–182.

(6) Thwe, M. M.; Liao, K. Durability of Bamboo-Glass Fiber Reinforced Polymer Matrix Hybrid Composites. *Compos Sci Technol* **2003**, *63* (3–4), 375–387.

(7) Cetinkaya, S.; Zhou, Q.; Zhang, S.; Mohebi, A.; Quach, D. V.; Stroeve, P.; Cetinkaya, S.; Zhou, Q.; Zhang, S.; Mohebi, A.; Quach, D. V.; Stroeve, P. Nucleation and Growth of Zinc Sulfide Nanoparticles in Ultrathin Polymer Films by Layer-by-Layer Polyionic Assemblies. *Soft Nanoscience Letters* **2011**, *1* (2), 33–40.

(8) Bourgeat-Lami, E.; Negrete Herrera, N.; Putaux, J. L.; Reculusa, S.; Perro, A.; Ravaine, S.; Mingotaud, C.; Duguet, E. Surface Assisted Nucleation and Growth of Polymer Latexes on Organically-Modified Inorganic Particles. *Macromol Symp* **2005**, *229* (1), 32–46.

(9) Laird, E. D.; Li, C. Y. Structure and Morphology Control in Crystalline Polymer-Carbon Nanotube Nanocomposites. *Macromolecules* **2013**, *46* (8), 2877–2891.

(10) Li, L.; Li, C. Y.; Ni, C. Polymer Crystallization-Driven, Periodic Patterning on Carbon Nanotubes. *J Am Chem Soc* **2006**, *128* (5), 1692–1699.

(11) Carrola, M.; Fallahi, H.; Koerner, H.; Pérez, L. M.; Asadi, A. Fundamentals of Crystalline Evolution and Properties of Carbon Nanotube-Reinforced Polyether Ether Ketone Nanocomposites in Fused Filament Fabrication. *ACS Appl Mater Interfaces* **2023**, *15* (18), 22506–22523.

(12) Trustrum, K.; Jayatilaka, A. D. S. Applicability of Weibull Analysis for Brittle Materials. *J Mater Sci* **1983**, *18* (9), 2765–2770.

(13) Weibull, W. A Statistical Distribution Function of Wide Applicability. *J Appl Mech* **1951**, 18, 293-297.

(14) Zhang, F.; Jiang, W.; Song, X.; Kang, J.; Cao, Y.; Xiang, M. Effects of Hyperbranched Polyester-Modified Carbon Nanotubes on the Crystallization Kinetics of Polylactic Acid. *ACS Omega* **2021**, *6* (15), 10362–10370.

(15) Lu, M.; Arias-Monje, P. J.; Ramachandran, J.; Gulgunje, P. V.; Luo, J.; Kirmani, M. H.; Meredith, C.; Kumar, S. Stabilization of Polyacrylonitrile Fibers with Carbon Nanotubes. *Polym Degrad Stab* **2021**, *188*, 109567.

(16) Franklin, R.; Xu, W.; Ravichandran, D.; Jambhulkar, S.; Zhu, Y.; Song, K. Reinforcing Carbonized Polyacrylonitrile Fibers with Nanoscale Graphitic Interface-Layers. *J Mater Sci Technol* **2021**, *95*, 78-87.

(17) Chang, H.; Luo, J.; Liu, H. C.; Zhang, S.; Park, J. G.; Liang, Z.; Kumar, S. Stabilization Study of Polyacrylonitrile/Cellulose Nanocrystals Composite Fibers. *ACS Appl Polym Mater* **2019**, *1* (5), 1015–1021.

(18) Chang, H.; Lu, M.; Luo, J.; Park, J. G.; Liang, R.; Park, C.; Kumar, S. Polyacrylonitrile/Boron Nitride Nanotubes Composite Precursor and Carbon Fibers. *Carbon* **2019**, *147*, 419–426.
